# Tyrosine phosphorylation of IRF3 by BLK facilitates its sufficient activation and innate antiviral response

**DOI:** 10.1371/journal.ppat.1011742

**Published:** 2023-10-23

**Authors:** Wei-Wei Li, Xu-Xu Fan, Zi-Xiang Zhu, Xue-Jing Cao, Zhao-Yu Zhu, Dan-Shi Pei, Yi-Zhuo Wang, Ji-Yan Zhang, Yan-Yi Wang, Hai-Xue Zheng

**Affiliations:** 1 State Key Laboratory for Animal Disease Control and Prevention, College of Veterinary Medicine, Lanzhou University, Lanzhou Veterinary Research Institute, Chinese Academy of Agricultural Sciences, Lanzhou, China; 2 Key Laboratory of Special Pathogens and Biosafety, Wuhan Institute of Virology, Center for Biosafety Mega-Science, Chinese Academy of Sciences, Wuhan, China; 3 Gansu Province Research Center for Basic Disciplines of Pathogen Biology, Lanzhou, China; University of Southern California, UNITED STATES

## Abstract

Viral infection triggers the activation of transcription factor IRF3, and its activity is precisely regulated for robust antiviral immune response and effective pathogen clearance. However, how full activation of IRF3 is achieved has not been well defined. Herein, we identified BLK as a key kinase that positively modulates IRF3-dependent signaling cascades and executes a pre-eminent antiviral effect. BLK deficiency attenuates RNA or DNA virus-induced ISRE activation, interferon production and the cellular antiviral response in human and murine cells, whereas overexpression of BLK has the opposite effects. BLK-deficient mice exhibit lower serum cytokine levels and higher lethality after VSV infection. Moreover, BLK deficiency impairs the secretion of downstream antiviral cytokines and promotes Senecavirus A (SVA) proliferation, thereby supporting SVA-induced oncolysis in an *in vivo* xenograft tumor model. Mechanistically, viral infection triggers BLK autophosphorylation at tyrosine 309. Subsequently, activated BLK directly binds and phosphorylates IRF3 at tyrosine 107, which further promotes TBK1-induced IRF3 S386 and S396 phosphorylation, facilitating sufficient IRF3 activation and downstream antiviral response. Collectively, our findings suggest that targeting BLK enhances viral clearance via specifically regulating IRF3 phosphorylation by a previously undefined mechanism.

## Author summary

Transcription factor IRF3-mediated type I interferon production is essential for antiviral innate immunity. Understanding how full activation of IRF3 is regulated is important to decipher the detailed mechanisms of IRF3-dependent signal transduction. Here, we report that BLK promotes IRF3 activation, leading to elevated antiviral cytokine production against multiple invading viruses both *in vivo* and *in vitro*. Upon viral infection, BLK undergoes Y309 autophosphorylation and directly phosphorylates IRF3 at Y107, together with TBK1-induced IRF3 S386 and S396 phosphorylation, to achieve sufficient IRF3 activation and elicit robust downstream antiviral responses. Overall, these data suggest that BLK is a critical effector of IRF3 phosphorylation. This study advances the understanding of the precise regulatory mechanisms of IRF3 activation. Manipulation of these mechanisms may help to develop novel therapeutics for infectious and inflammatory diseases.

## Introduction

The innate immune system is the first line of host defense against microbial infection [1,2]. Upon infection, structurally conserved microbial components called pathogen-associated molecular patterns (PAMPs) are detected by cellular pattern-recognition receptors (PRRs), which trigger a series of intracellular immune signaling cascades for the ultimate eradication of invading pathogens and clearance of infected cells [3,4]. Among the identified PRRs, retinoic acid-inducible gene-I (RIG-I)-like receptors (RLRs), including RIG-I and melanoma differentiation-associated gene 5 (MDA5), mainly detect cytosolic viral RNA, and cyclic GMP-AMP synthase (cGAS) primarily senses cytoplasmic viral DNA, whereas membrane-associated Toll-like receptor 3 (TLR3) recognizes extracellular and endosomal viral RNA [5–8]. Once viral nucleic acids are sensed, PRRs recruit different adaptor proteins, such as virus-induced-signaling adapter (VISA), stimulator of interferon genes (STING), or TIR domain-containing adapter molecule 1 (TICAM1) [9–11]. Aforementioned adaptors then act as a central platform for recruitment and activation of a common downstream kinase TBK1. Activated TBK1 further phosphorylates interferon regulatory factor 3 (IRF3) and triggers its homodimerization and nuclear translocation, ultimately leading to the induction of a wide range of downstream antiviral effectors and the establishment of an antiviral state [12–14]. However, the precise mechanisms underlying how IRF3 is recruited into the adaptor signalosome remain largely unknown.

The host innate antiviral response should be of an appropriate duration and magnitude to efficiently eliminate the invading viruses while avoiding undesirable damage [15]. Abundant evidence suggests that posttranslational modifications play a critical role in regulating virus-triggered type I interferon (IFN) induction [16]. As a key signal adaptor and transcription factor, IRF3 activity is strictly regulated by multiple posttranslational modifications, including phosphorylation, deSUMOylation, ubiquitination and acetylation [17–20]. Well-presented studies have reported that the upstream kinase TBK1 phosphorylates IRF3 at S386 and S396, and IKKε phosphorylates IRF3 at S396, which are required for IRF3 dimerization and activation. However, TBK1 and IKKε are necessary but not sufficient for IRF3 activation [21–24]. Although there are many reports on host factors regulating the activation or degradation of IRF3, current understanding regarding which mediators drive TBK1-induced phosphorylation of IRF3 and how the status of IRF3 activation is controlled is still limited. Understanding the link between phosphorylation and activation of IRF3 has been a topic of interest in the innate immunity field.

Src family kinases (SFKs) are the largest non-receptor tyrosine kinase family with nine members, including SRC, HCK, LYN, FYN, FGR, LCK, YES, YLK, and BLK [25]. All SFK members share considerable homology in domain structure and exhibit partial overlaps in function, but their cell expression lineages are different [26, 27]. Previous studies have shown that B lymphoid tyrosine kinase (BLK) is primarily expressed in B cells [28]. However, recent expression pattern analysis found that BLK is also expressed in a variety of murine cell types outside of the B cell lineage, including immune and non-immune cells, such as plasmacytoid dendritic cells (pDCs), pancreatic β cells, epithelial cancer cells, bone marrow progenitor cells, early thymus precursors, interleukin-17-producing γδ T cells, CD4^-^ CD8^-^ αβ T cells, etc [29–31]. It has been shown that BLK-mediated cGAS phosphorylation at tyrosine 215 facilitates the cytosolic retention of cGAS to suppress DNA damage and tumorigenesis [32]. Genome-wide association studies have found that multiple single nucleotide polymorphisms (SNPs) in the BLK locus are associated with autoimmune disorders in several populations, including systemic lupus erythematosus, rheumatoid arthritis, systemic sclerosis, and so forth. It is largely unknown how SNP risk alleles of BLK or its aberrant expression eventually causes autoimmune diseases [33–35]. Structurally, BLK consists of N-terminal Src homology (SH) 2 and 3 domains and a C-terminal protein kinase domain. The K269 site in the protein kinase domain is responsible for binding ATP, which is essential for its catalytic activity [36]. Although tyrosine phosphorylation accounts for only ~2.5% of total protein phosphorylation, it exhibits profound effects on cellular physiology [37]. Nevertheless, the involvement of tyrosine phosphorylation in innate antiviral response is not fully understood, particularly in the classical antiviral pathways.

In this study, we identified BLK as a positive modulator of IRF3-dependent antiviral signaling in response to viral infection. *In vivo* and *in vitro* assays further indicated that BLK interacted with IRF3 and mediated its Y107 phosphorylation to promote sufficient activation of IRF3. Taken together, our observations demonstrate that BLK-mediated phosphorylation of IRF3 is essential for eliciting innate antiviral response and provide new mechanistic insights into IRF3 activation.

## Results

### BLK positively regulates the innate immune response against viral infection

**Fig 1:**
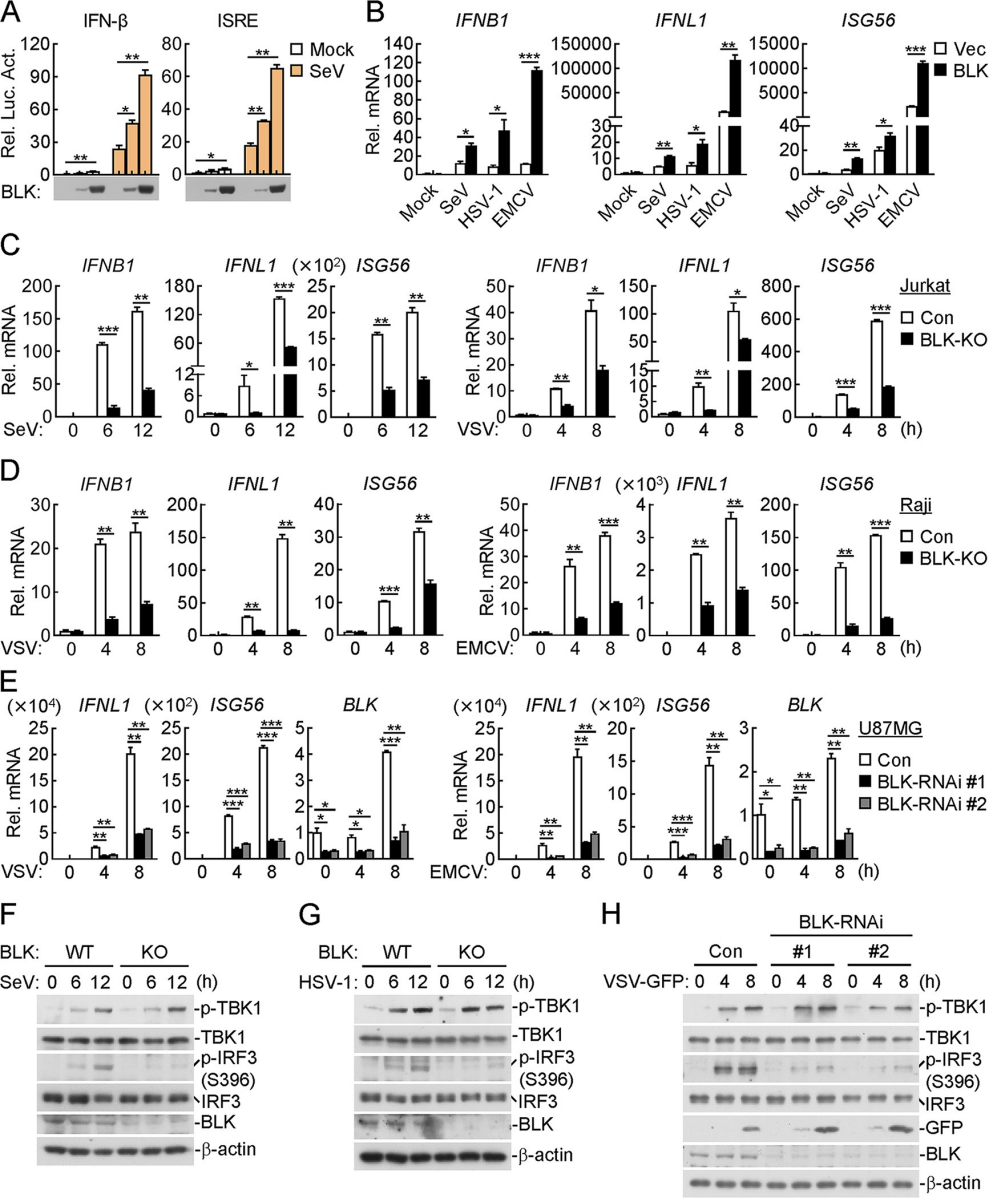
Identification of BLK as a positive regulator of RNA or DNA virus-triggered signaling. **(A)** Effects of BLK on SeV-induced activation of IFN-β and ISRE. U87MG cells (1 × 10^5^) were co-transfected with the indicated reporter (IFN-β (0.05 μg), ISRE (0.05 μg)), pRL-TK (*Renilla* luciferase) reporter (0.01 μg) and increased amounts of BLK expression plasmids (0.01, 0.05 μg) for 24 h. Cells were then uninfected or infected with SeV (MOI, 1) for 10 h before luciferase assays. **(B)** Effects of BLK on SeV-, HSV-1- or EMCV-induced transcription of downstream antiviral genes. Raji cells were transduced with vector (Vec) or BLK expression plasmids by lentivirus-mediated gene transfer to establish the stable cell lines. Cells (2 × 10^5^) were then uninfected or infected with viruses (MOI, 1) for 8 h before qPCR analysis. **(C and D)** Effects of BLK deficiency on SeV-, VSV- or EMCV-induced transcription of downstream antiviral genes. Jurkat (C) or Raji (D) cells were transduced with control (Con) or the indicated gRNA plasmids targeting BLK by the CRISPR/Cas9 method to establish the stable cell lines. BLK-deficient and control cells (2 × 10^5^) were then uninfected or infected with viruses (MOI, 1) for the indicated times before qPCR analysis. **(E)** Effects of BLK knockdown on VSV- or EMCV-induced transcription of downstream antiviral genes. U87MG cells (2 × 10^5^) were transfected with the indicated siRNA (final concentration, 40 nM). Forty-eight hours later, cells were uninfected or infected with viruses (MOI, 1) for the indicated times before qPCR analysis. **(F and G)** Effects of BLK deficiency on SeV- or HSV-1-induced phosphorylation of TBK1 and IRF3. BLK-deficient and control Jurkat (F) or Raji (G) cells (2 × 10^5^) were infected with viruses (MOI, 1) for the indicated times before immunoblot analysis. **(H)** Effects of BLK knockdown on VSV-induced phosphorylation of TBK1 and IRF3. U87MG cells (2 × 10^5^) were transfected with the indicated siRNA (final concentration, 40 nM). Forty-eight hours later, cells were uninfected or infected with VSV-GFP (MOI, 1) for the indicated times before immunoblot analysis. Graphs show mean ± SD (*n* = 2 biological replicates in A, *n* = 2 technical replicates in B-E) from one representative experiment. Data are representative of at least three independent experiments with similar results. *P < 0.05, **P < 0.01, ***P < 0.001 (unpaired, two-tailed Student’s *t*-test).

To identify potential regulatory genes required for virus-triggered production of type I IFNs, we screened a kinase library containing 352 independent human cDNA clones for their abilities to regulate interferon-stimulated response element (ISRE) activity by reporter assays and identified BLK as a candidate protein. Reporter assays indicated that overexpression of BLK activated ISRE and IFN-β promoters and dose-dependently potentiated SeV-triggered ISRE and IFN-β activation, whereas BLK showed little effect on IFN-β-induced STAT1/2 and IFN-γ-induced IRF1 activation (Figs 1A, S1A, and S1B). Consistently, quantitative PCR (qPCR) analysis indicated that overexpression of BLK promoted the transcription of downstream genes, including *IFNB1*, *IFNL1* and *ISG56*, in response to SeV, EMCV, or HSV-1 infection or cytoplasmic transfection of high-molecular-weight (HMW) poly(I:C), which is mainly sensed by MDA5, in Jurkat, Raji or U87MG cell lines, respectively (Figs 1B, S1C and S1D). However, overexpression of BLK had no marked effects on IFN-β- and IFN-γ-induced transcription of downstream genes (S1E Fig). Moreover, the expression levels of BLK in the above cell lines were also detected (S1F–S1H Fig). These results suggest that BLK is involved in RNA or DNA virus-triggered induction of downstream antiviral genes.

Next, we determined whether endogenous BLK is necessary for virus-triggered signaling. We generated BLK-deficient Jurkat, Raji and U87MG cells by the CRISPR/Cas9 method. The transcription of downstream genes such as *IFNB1*, *IFNL1* and *ISG56* induced by SeV, VSV, EMCV, HSV-1, HCMV or transfected low-molecular-weight (LMW) poly(I:C), which is mostly recognized by RIG-I, or HMW poly(I:C) was markedly impaired in BLK-deficient Jurkat and Raji cells compared with that of control cells (Figs 1C, 1D, S2A and S2B). To further characterize the physiological function of BLK in nucleic acid-induced responses, we used a knockdown strategy involving two distinct siRNAs targeting the human *BLK* gene. qPCR assays indicated that BLK knockdown significantly attenuated the transcription of downstream genes such as *IFNL1* and *ISG56* induced by SeV, VSV, EMCV or transfected HMW or LMW poly(I:C) in U87MG cells (Figs 1E and S2C). Similarly, Senecavirus A (SVA)-induced transcription of downstream genes was also clearly impaired in BLK-deficient U87MG cells (S2D Fig). Intriguingly, BLK deficiency or knockdown dramatically inhibited SeV-, VSV-, EMCV- and HSV-1-triggered phosphorylation of IRF3 but not TBK1 in different cell types, which implied that BLK might function downstream of TBK1 (Figs 1F–1H and S2E). In addition, we noticed that BLK knockdown facilitated the replication of GFP-tagged VSV (Fig 1H). These results suggest that BLK is an important mediator of antiviral gene induction against viral infection.

### Blk deficiency attenuates virus-triggered innate antiviral signaling in murine cells

**Fig 2:**
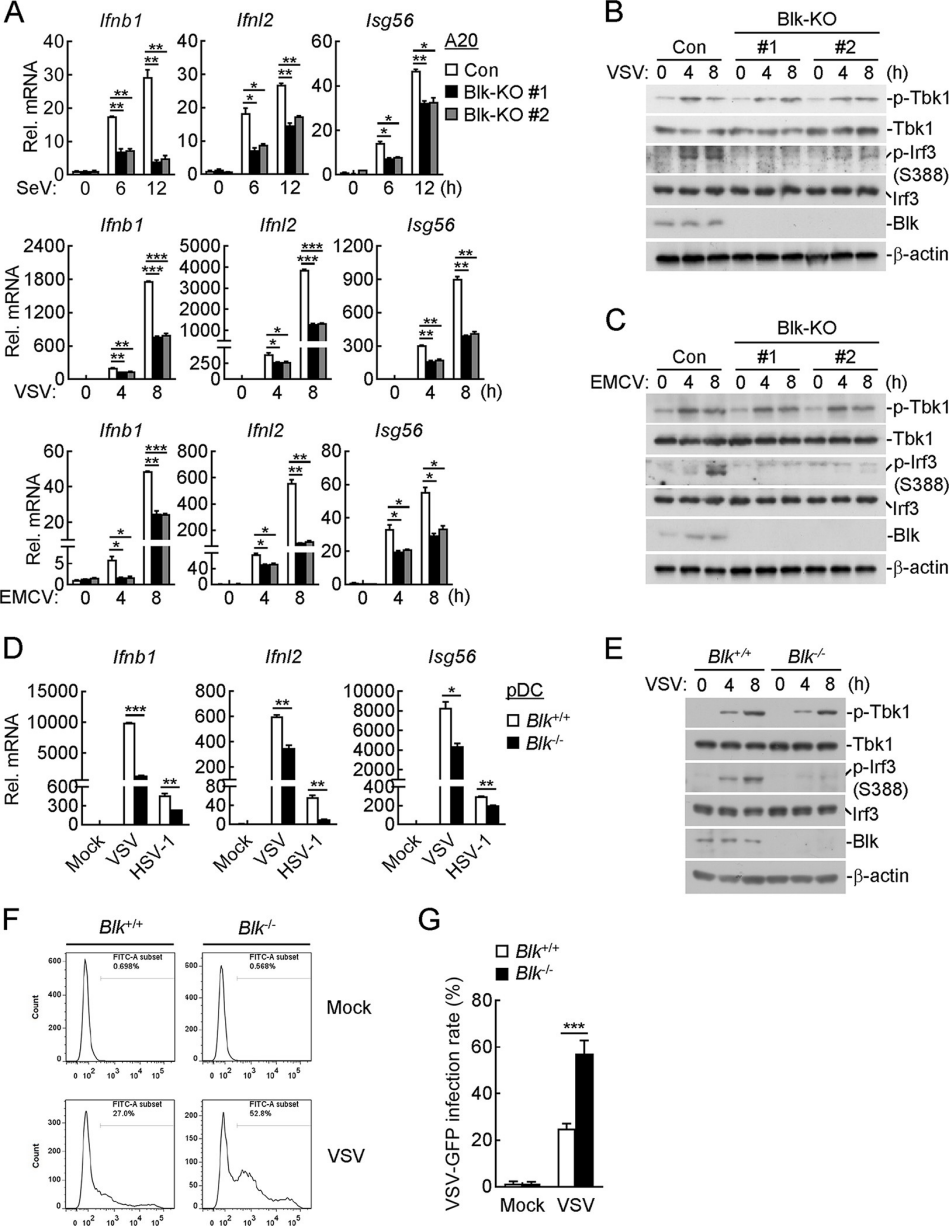
Blk is required for RNA or DNA virus-induced innate antiviral response in murine cells. **(A-C)** Effects of Blk deficiency on SeV-, VSV- or EMCV-induced transcription of downstream genes and phosphorylation of TBK1 and IRF3. A20 cells were transduced with control (Con) or the indicated gRNA plasmids targeting Blk by the CRISPR/Cas9 method to establish the stable cell lines. Blk-deficient and control cells (2 × 10^5^) were then uninfected or infected with viruses (MOI, 1) for the indicated times before qPCR (A) and immunoblot (B and C) analyses. **(D and E)** Effects of Blk deficiency on VSV- or HSV-1-induced transcription of downstream genes and phosphorylation of TBK1 and IRF3. pDCs derived from the spleens of *Blk*^+/+^ and *Blk*^-/-^ mice (1 × 10^5^) were infected with VSV or HSV-1 (MOI, 0.5) for 8 h before qPCR analysis (D) or infected with VSV (MOI, 0.5) for the indicated times before immunoblot analysis (E). **(F and G)** Effects of Blk deficiency on the replication of VSV. (F) pDCs derived from the spleens of *Blk*^+/+^ and *Blk*^-/-^ mice (1 × 10^5^) were infected with VSV-GFP (MOI, 0.2) for 8 h before flow cytometry analysis. (G) Statistical analysis in the form of a bar graph for the data in F. Graphs show mean ± SD (*n* = 2 technical replicates in A and D, *n* = 3 biological replicates in G) from one representative experiment. Data are representative of at least three independent experiments with similar results. *P < 0.05, **P < 0.01, ***P < 0.001 (unpaired, two-tailed Student’s *t*-test).

We next generated Blk-deficient A20 cells, an immortalized murine B lymphoma cell line, and investigated the physiological functions of Blk in murine cells. As shown in Fig 2A, Blk deficiency attenuated SeV-, VSV- and EMCV-induced transcription of *Ifnb1*, *Ifnl2* and *Isg56* genes. Consistent with virus-induced phosphorylation changes of IRF3 and TBK1 in human cells, Blk deficiency evidently dampened VSV- and EMCV-triggered phosphorylation of IRF3 but not TBK1 in A20 cells (Fig 2B and 2C).

To further confirm the roles of Blk in virus-triggered innate immune signaling, Blk-deficient mice were generated using the CRISPR/Cas9 method (S3A Fig). The successful deletion of *Blk* in the knockout mice was verified by genotyping analysis (S3B Fig). pDCs function as sentinels of viral infection by producing copious amounts of type I and III IFNs in response to pathogenic stimuli [38]. pDCs potently initiate the TBK1-IRF3 axis following virus-derived nucleic acid recognition predominantly via endosomal TLR7/9 or cytoplasmic RIG-I/cGAS sensors [39, 40]. RNA interference-mediated IRF3 knockdown experiments have established IRF3 as a crucial mediator of type I IFN induction in pDCs [39, 41]. Nonetheless, the putative regulatory role of Blk in signaling induced by antiviral sensors of pDCs has not been investigated. We firstly isolated pDCs from the spleens of *Blk*^-/-^ mice and their wild-type littermates, and then infected these cells with VSV and HSV-1. As shown in Fig 2D and 2E, Blk deficiency impaired VSV- and HSV-1-induced transcription of downstream genes, including *Ifnb1*, *Ifnl2* and *Isg56*, and phosphorylation of IRF3 but not TBK1. Consistently, flow cytometry analysis demonstrated that GFP-tagged VSV replication was enhanced in Blk-deficient pDCs in comparison with that of wild-type cells (Fig 2F and 2G). Altogether, these data suggest that Blk is required for virus-triggered downstream antiviral signaling in murine cells.

### Blk is essential for innate antiviral response *in vivo*

**Fig 3:**
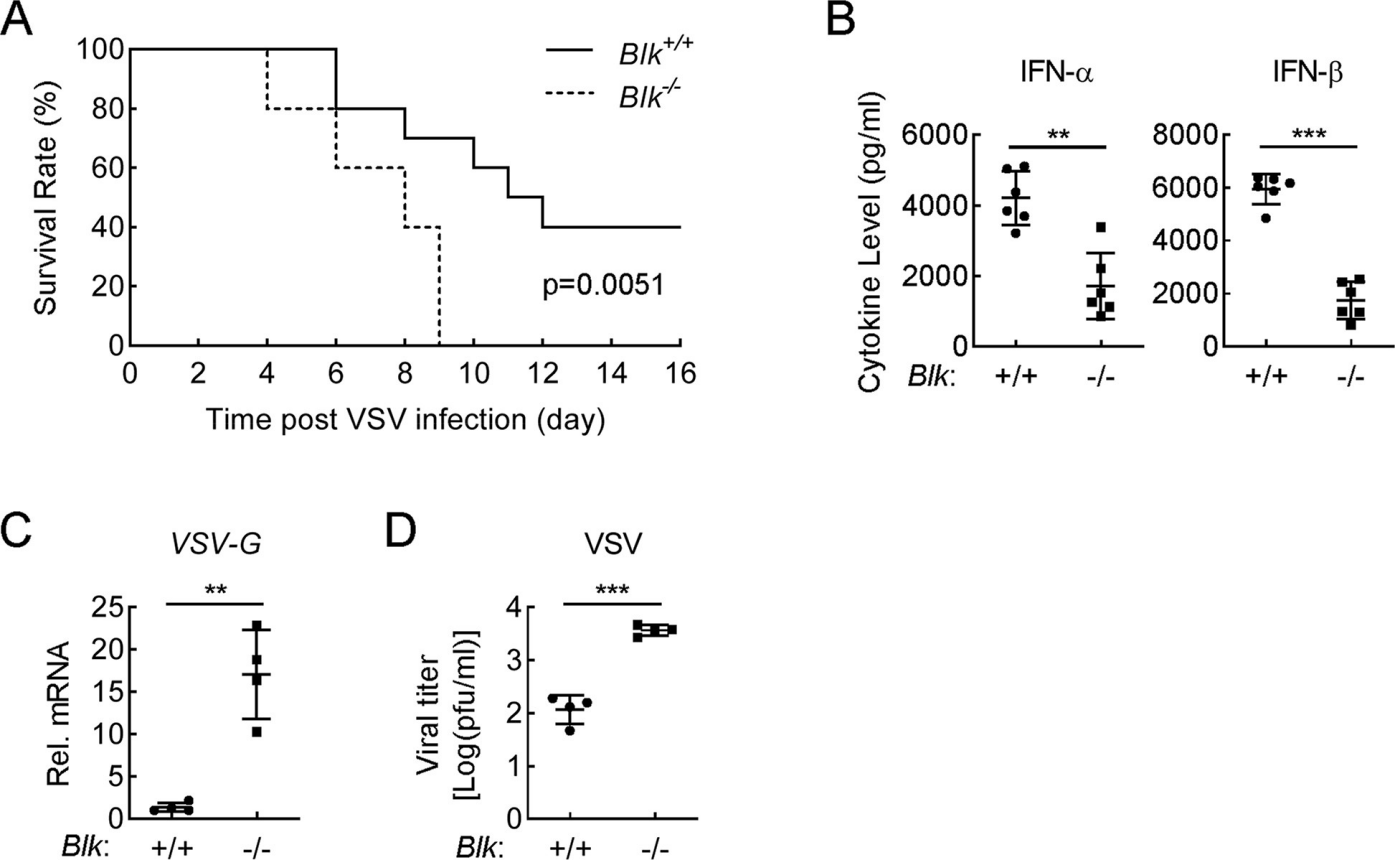
BLK is critical for host defense against VSV infection in mice. **(A)** Effects of Blk deficiency on VSV-induced death in mice. Sex- and age-matched *Blk*^+/+^ and *Blk*^-/-^ mice (n = 10 for each group) were infected intraperitoneally with VSV (1 × 10^8^ PFU per mouse), and mouse survival was recorded daily for 16 days. **(B)** Effects of Blk deficiency on VSV-induced serum levels of IFN-α and IFN-β. Sex- and age-matched *Blk*^+/+^ and *Blk*^-/-^ mice (n = 6 for each group) were infected intraperitoneally with VSV (1 × 10^8^ PFU per mouse) for 6 h before serum cytokines were measured by ELISA. **(C and D)** Effects of Blk deficiency on viral replication in the spleens of VSV-infected mice. Sex- and age-matched *Blk*^+/+^ and *Blk*^-/-^ mice (n = 4 for each group) were infected intraperitoneally with VSV (1 × 10^8^ PFU per mouse) for 4 days. Viral titers and genomic copy numbers in the spleens of VSV-infected mice were quantified by qPCR (C) and plaque (D) assays, respectively. Graphs show mean ± SD from one representative experiment. Data are representative of at least two independent experiments with similar results. **P < 0.01, ***P < 0.001 (unpaired, two-tailed Student’s *t*-test). For the mouse survival study in A, Kaplan-Meier survival curves were generated and analyzed by the log-rank test.

To evaluate the importance of Blk in host defense against viral infection *in vivo*, age- and sex-matched *Blk*^-/-^ and *Blk*^+/+^ mice were infected with VSV by intraperitoneal (i.p.) injection. As shown in Fig 3A, compared with wild-type mice, *Blk*^-/-^ mice were more susceptible to VSV-induced death. Moreover, VSV-induced levels of serum cytokines including IFN-α and IFN-β in *Blk*^-/-^ mice were obviously lower than those of wild-type mice (Fig 3B). Consistently, the mRNA levels and viral titers of VSV in the spleen were much higher in *Blk*^-/-^ mice in comparison to those of wild-type mice (Fig 3C and 3D). Collectively, these results suggest that Blk is necessary for efficient host defense against VSV infection in mice.

### BLK deficiency enhances SVA-induced oncolysis by inhibiting cellular antiviral response to promote SVA proliferation

SVA causes vesicular disease and epidemic transient neonatal losses in pigs but is non-pathogenic in humans and mice [42, 43]. Recently, SVA was identified as a potent oncolytic picornavirus with cytolytic activity due to its ability to penetrate solid tumors and spread rapidly through the vascular system, subsequently inducing cytotoxicity and contributing to the amplified cell killing by infecting additional tumor cells [44–46]. Moreover, SVA has a highly selective tropism for tumor cells with neuroendocrine characteristics [47, 48]. Treatment with SVA via intravenous administration is able to eliminate approximately 80% of tumor cells and shows prospective preclinical efficacy in athymic mice bearing xenograft tumors, which presents an attractive therapeutic approach for cancer [49, 50]. U87MG, a neuroendocrine tumor cell, is capable of forming tumor in athymic mice and is widely used as an *in vivo* xenograft tumor model [51–53]. As shown in S2D Fig, our results manifested that BLK deficiency obviously impaired SVA-induced transcription of downstream antiviral genes in U87MG cells. Therefore, we wondered whether BLK indeed modulates the production of downstream antiviral genes to affect SVA replication.

**Fig 4:**
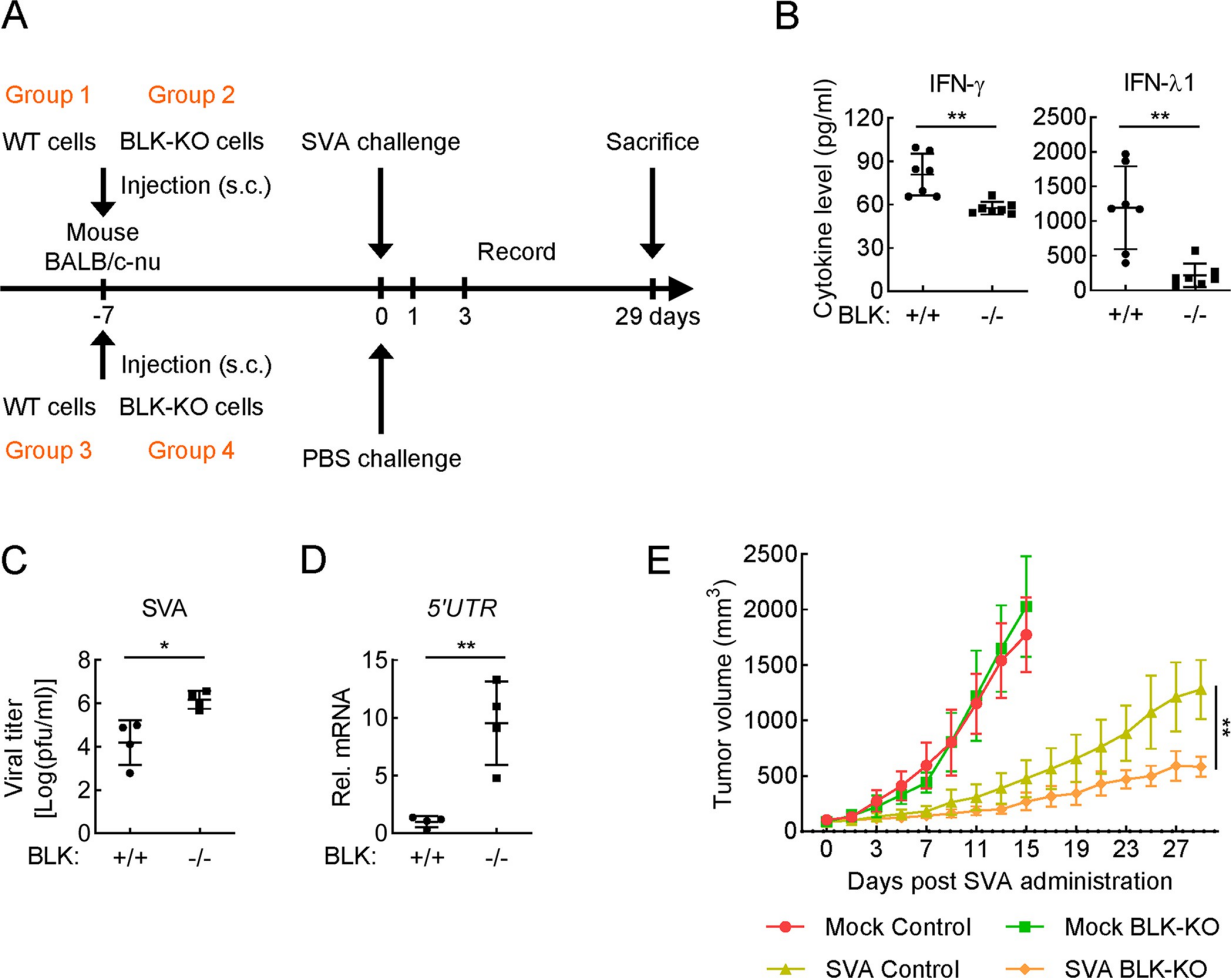
BLK is essential for host defense against SVA infection in the U87MG xenograft tumor model. **(A)** Schematic diagram of the SVA efficacy experiment *in vivo*. **(B)** Effects of BLK deficiency on SVA-induced cytokine levels in U87MG xenograft tumors. Athymic female mice (BALB/c-nu) bearing wild-type or BLK-deficient U87MG tumors (n = 7 for each group) were challenged with SVA (1 × 10^8^ PFU per mouse) for 24 h via a single intravenous delivery. Subsequently, tumors were excised and subjected to ELISA measurement. **(C and D)** Effects of BLK deficiency on SVA replication kinetics in U87MG xenograft tumors. Athymic female mice (BALB/c-nu) bearing wild-type or BLK-deficient U87MG tumors (n = 4 for each group) were challenged with SVA (1 × 10^8^ PFU per mouse) for 3 days via a single intravenous delivery. Subsequently, viral titers and genomic copy numbers in tumor tissues were quantified by plaque (C) and qPCR (D) assays, respectively. 5’UTR is the N-terminal noncoding region of the SVA genome. **(E)** Effects of BLK deficiency on SVA-induced *in vivo* antitumor efficacy. Athymic mice bearing pre-established U87MG xenograft tumors within each cohort were randomly distributed (n = 3 for each group) and challenged with SVA or PBS at a dose of 1 × 10^8^ PFU per mouse into the lateral tail vein. Antitumor efficacy was determined by measuring tumor volumes every 2 days following SVA administration. Graphs show mean ± SD from one representative experiment. Data are representative of at least two independent experiments with similar results. *P < 0.05, **P < 0.01 (unpaired, two-tailed Student’s *t*-test).

We next evaluated the role of BLK in host defense against viral infection based on an *in vivo* xenograft tumor model. Briefly, wild-type or BLK-deficient U87MG cells in 10% phenol red-free Matrigel were injected subcutaneously into the flank of athymic female mice. Once tumors reached volumes of approximately 100 mm^3^ (~ 7 days), mice within each cohort were randomly distributed and challenged with SVA or PBS via a single intravenous delivery. Twenty-four hours after SVA challenge, tumors were excised and subjected to ELISA for cytokine quantification. Three days after SVA challenge, tumors were excised and subjected to plaque and qPCR assays. Furthermore, *in vivo* antitumor efficacy was determined by measuring tumor volumes every 2 days following SVA administration (Fig 4A). The detailed results are as follows. As shown in Fig 4B, the cytokines induced by SVA infection, including IFN-γ and IFN-λ1, were severely impaired in BLK^-/-^ tumors compared with that of wild-type tumors. Moreover, BLK^-/-^ tumor tissues infected with SVA for 3 days showed much higher viral titers and genomic copies than those of infected wild-type tumor tissues (Fig 4C and 4D). Additionally, we performed an *in vivo* SVA efficacy evaluation in athymic mice bearing U87MG xenograft tumors via intravenous delivery of SVA or PBS. As shown in Fig 4E, U87MG tumors derived from the control mice (groups 3 and 4) that were injected with PBS grew rapidly, whereas the volumes of wild-type (group 3) and BLK-deficient (group 4) U87MG tumors were not significantly different. Compared with the control groups, both groups of mice challenged with SVA (groups 1 and 2) showed profoundly delayed tumor progression. Notably, the tumor growth rate was impaired in BLK-deficient group (group 2) in comparison to wild-type group (group 1). These results suggest that BLK deficiency attenuates the secretion of IFNs and promotes SVA replication, thereby facilitating SVA-mediated antitumor efficacy in athymic mice bearing U87MG xenograft tumors.

### BLK binds directly to IRF3

**Fig 5:**
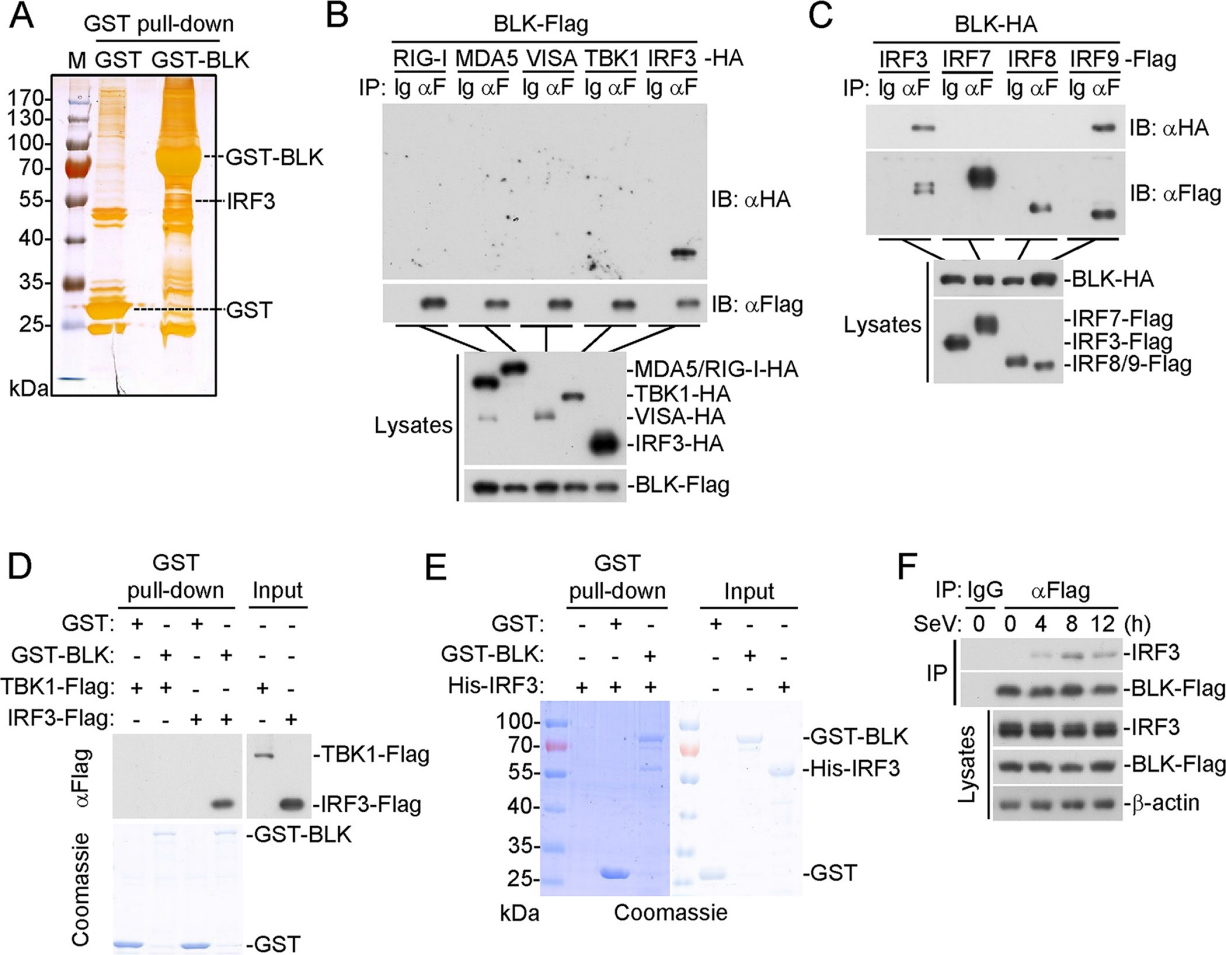
BLK associates with IRF3. **(A)** Identification of the BLK interactome by silver staining and mass spectrometry methods. Silver staining showing GST-associated factors (Control) and GST-BLK-associated factors. IRF3 was one of the convincing interacting factors. **(B)** BLK interacts with IRF3 in the mammalian overexpression system. HEK293 cells (2 × 10^6^) were transfected with the indicated plasmids for 24 h. Coimmunoprecipitation and immunoblot analysis were performed with the indicated antibodies. **(C)** BLK interacts with IRF3 and IRF9. Coimmunoprecipitation and immunoblot analysis were similarly performed as in B. **(D)** BLK associates with IRF3. Prokaryotically expressed and purified GST-BLK protein coupled to glutathione sepharose beads was incubated with purified TBK1-Flag or IRF3-Flag protein for 3 h at 4°C and then subjected to *in vitro* GST pull-down assays. GST: glutathione S-transferase. **(E)** BLK directly binds to IRF3. Prokaryotically expressed and purified His-IRF3 and GST-BLK proteins were subjected to *in vitro* GST pull-down assays. **(F)** BLK associates with endogenous IRF3 upon SeV infection. Jurkat cells stably expressing BLK-Flag (2 × 10^7^) were uninfected or infected with SeV (MOI, 1) for the indicated times. Coimmunoprecipitation and immunoblot analysis were performed with the indicated antibodies. Data are representative of at least three independent experiments with similar results.

We next investigated the mechanism by which BLK exerts its function in virus-triggered innate antiviral response. To uncover the potential interaction networks, we first characterized the BLK interactome utilizing silver staining and mass spectrometry methods. Notably, IRF3 was among the convincing interactors (Fig 5A and S1 Table). Coimmunoprecipitation experiments further confirmed that BLK was associated with IRF3 but not RIG-I, MDA5, VISA, or TBK1 in the mammalian overexpression system (Fig 5B). In similar coimmunoprecipitation experiments, BLK was associated with IRF9 but not IRF7 or IRF8 (Fig 5C). Furthermore, *in vitro* pull-down assays using purified recombinant BLK and cell lysates transfected with TBK1 or IRF3 showed that BLK bound to IRF3 but not TBK1 (Fig 5D). Moreover, further pull-down assays using prokaryotically expressed BLK and IRF3 showed that BLK directly interacted with IRF3 (Fig 5E). To explore the endogenous interaction between BLK and IRF3, we established a Jurkat cell line stably expressing BLK. After SeV infection, endogenous IRF3 interacted with BLK (Fig 5F). Altogether, these data suggest that BLK directly interacts with IRF3.

### BLK catalyzes the phosphorylation of IRF3 at tyrosine 107

**Fig 6:**
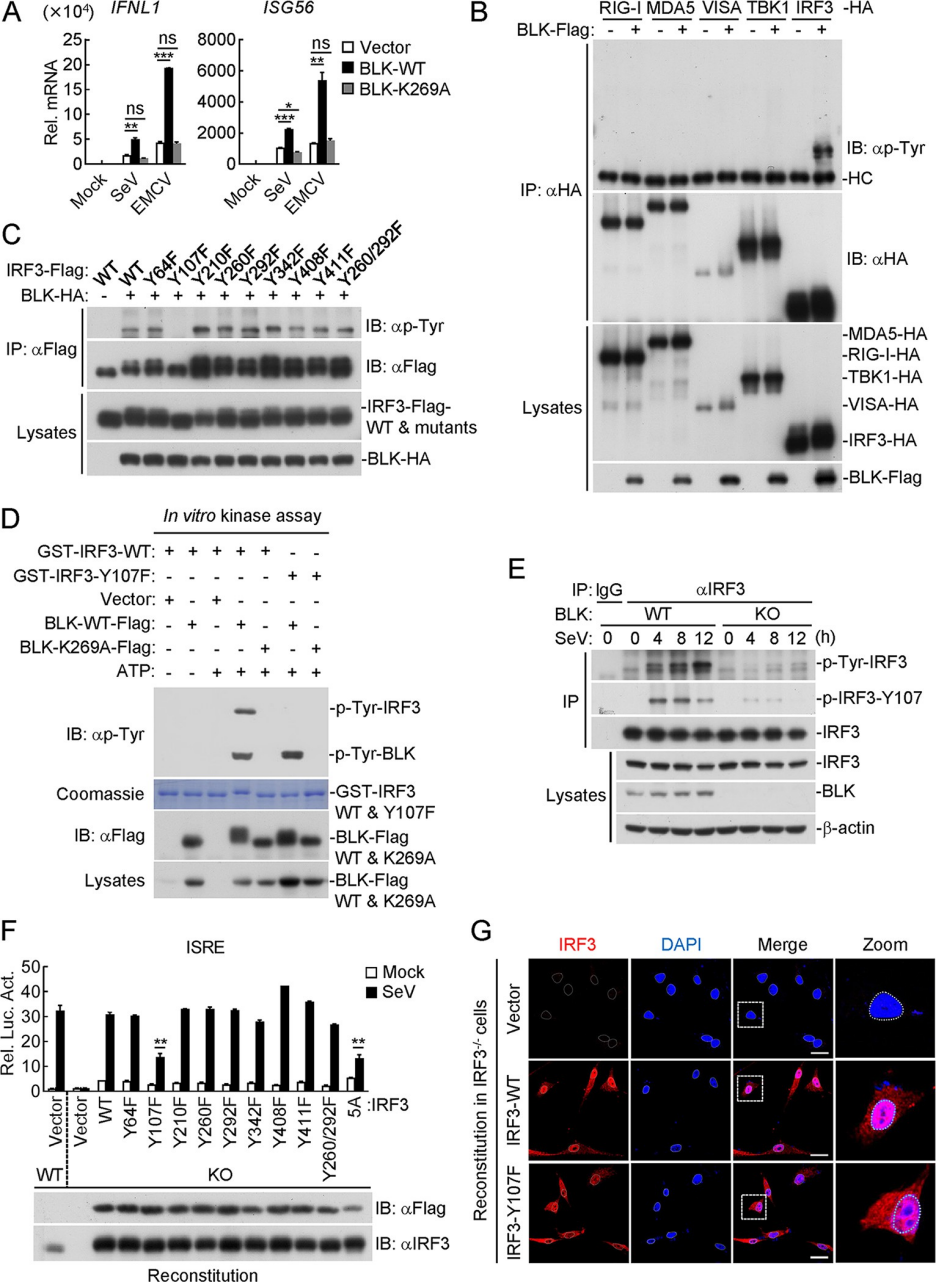
Phosphorylation of IRF3 at Y107 mediated by BLK is essential for virus-triggered innate antiviral response. **(A)** Effects of BLK and BLK(K269A) on SeV- or EMCV-induced transcription of downstream antiviral genes. U87MG cells (2 × 10^5^) were transfected with BLK or BLK(K269A) plasmids for 24 h. Cells were then uninfected or infected with viruses (MOI, 1) for 8 h before qPCR analysis. **(B)** BLK mediates tyrosine phosphorylation of IRF3. HEK293 cells (2 × 10^6^) were transfected with the indicated plasmids for 24 h. Coimmunoprecipitation and immunoblot analysis were performed with the indicated antibodies. **(C)** BLK phosphorylates IRF3 at Y107. HEK293 cells (2 × 10^6^) were transfected with the indicated plasmids for 24 h. Coimmunoprecipitation and immunoblot analysis were performed with the indicated antibodies. **(D)** BLK phosphorylates IRF3 but not IRF3-Y107F. Purified Flag-tagged BLK and BLK(K269A) coupled with Protein G sepharose beads were subjected to *in vitro* kinase assays with purified GST-tagged IRF3 or IRF3-Y107F. “Vector” here means the control Protein G sepharose beads. **(E)** Effects of BLK deficiency on SeV-triggered IRF3 Y107 phosphorylation. BLK-deficient and control U87MG cells (2 × 10^7^) were uninfected or infected with SeV (MOI, 1) for the indicated times. Coimmunoprecipitation and immunoblot analysis were performed with the indicated antibodies. **(F)** Effects of IRF3 or its mutant recovery on SeV-induced ISRE activation. U87MG cells were transduced with gRNA plasmids targeting IRF3 by the CRISPR/Cas9 method to establish the stable cell lines with puromycin (1 μg/mL) selection. IRF3-deficient and control U87MG cells (1 × 10^5^) were co-transfected with ISRE reporter (0.05 μg), pRL-TK reporter (0.01 μg), and either IRF3 or its mutant (0.05 μg) plasmids for 24 h and then uninfected or infected with SeV (MOI, 1) for 10 h before luciferase assays. The lower blots show the expression levels of IRF3 and its mutants as detected by anti-Flag or anti-IRF3 antibodies, respectively. **(G)** Effects of IRF3 or IRF3(Y107F) recovery on SeV-induced IRF3 nuclear translocation. IRF3-deficient U87MG cells were transduced with vector, IRF3, or IRF3(Y107F) plasmids by lentivirus-mediated gene transfer to establish the stable cell lines with blasticidin S (10 μg/mL) selection. Subsequently, the indicated cell lines (1 × 10^5^) were infected with SeV (MOI, 1) for 4 h, then fixed with 4% paraformaldehyde and stained with anti-IRF3 antibody before confocal microscopy. Scale bars, 50 μm. Graphs show mean ± SD (*n* = 2 technical replicates in A, *n* = 2 biological replicates in F) from one representative experiment. Data are representative of at least three independent experiments with similar results. *P < 0.05, **P < 0.01, ***P < 0.001, ns, not significant (unpaired, two-tailed Student’s *t*-test).

BLK is a crucial member of SFKs, and its catalytic activity mainly depends on the K269 residue, which is responsible for ATP binding [36, 54]. We wondered whether the kinase activity of BLK is indispensable for its modulation of virus-triggered innate antiviral signaling. As shown in Figs 6A and S4A, overexpression of BLK significantly potentiated SeV- and EMCV-induced transcription of *IFNL1* and *ISG56* genes, whereas BLK(K269A), a kinase-defective mutant due to the lysine to alanine substitution in the ATP-binding domain, showed little effect. Moreover, BLK enhanced tyrosine phosphorylation of IRF3 but not other related adaptors in the mammalian overexpression system (Figs 6B and S4B). To map potential residues of IRF3 that are modified by BLK, we next individually mutated all 8 tyrosine residues within IRF3 to phenylalanine. As shown in Fig 6C, BLK could enhance tyrosine phosphorylation of all IRF3 mutants except IRF3(Y107F). Remarkably, co-transfection of BLK and IRF3 resulted in a migration of IRF3 towards higher molecular weight, with the shifted protein band representing the phosphorylated IRF3 (Fig 6C). *In vitro* kinase assays indicated that BLK catalyzed tyrosine phosphorylation of IRF3 but not IRF3(Y107F), and BLK(K269A) could not catalyze tyrosine phosphorylation of IRF3 or IRF3(Y107F) (Fig 6D). To determine whether BLK indeed targets IRF3 Y107 for phosphorylation, we further generated an antibody that specifically recognizes the IRF3 Y107-phosphorylated epitope. As shown in Fig 6E, BLK deficiency markedly impaired SeV-induced total tyrosine phosphorylation as well as Y107 phosphorylation of endogenous IRF3. Collectively, these results suggest that BLK directly phosphorylates IRF3 at Y107.

We next asked whether BLK-mediated phosphorylation of IRF3 is indispensable for its role in virus-triggered innate antiviral signaling. Reporter assays indicated that reconstitution of IRF3-deficient cells with all IRF3 mutants except IRF3(Y107F) restored SeV-induced ISRE activation well (Fig 6F). Of note is that IRF3(Y107F) activated ISRE at levels comparable to IRF3(5A) after SeV infection. IRF3(5A) serves as a negative control here, in which the five serine residues 396, 398, 402, 404, and 405 are all mutated to alanine (A) (Fig 6F). Additionally, confocal microscopy experiments indicated that SeV-induced nuclear translocation of IRF3 was impaired in IRF3(Y107F)-reconstituted cells in comparison to IRF3-reconstituted cells (Fig 6G). Statistical analysis of IRF3 fluorescence intensity in the cytoplasm and nucleus also confirmed the above results (S4C Fig). Intriguingly, sequence analysis found that Y107 of IRF3 was conserved in various species (S4D Fig). Therefore, we next investigated whether Y107 of Irf3 has similar roles in murine cells. As shown in S4E Fig, Irf3 deficiency dramatically impaired VSV-induced transcription of *Ifnb1* and *Isg56* genes in A20 cells. Reconstitution of Irf3-deficient cells with wild-type Irf3 but not Irf3(Y107F) restored VSV-induced transcription of downstream antiviral genes. Taken together, these results suggest that BLK catalyzes tyrosine phosphorylation of human and murine IRF3 at Y107, which is necessary for IRF3 to regulate virus-triggered innate antiviral signaling.

### Autophosphorylation of BLK at Y309 is essential for its regulation of IRF3-dependent signaling

**Fig 7:**
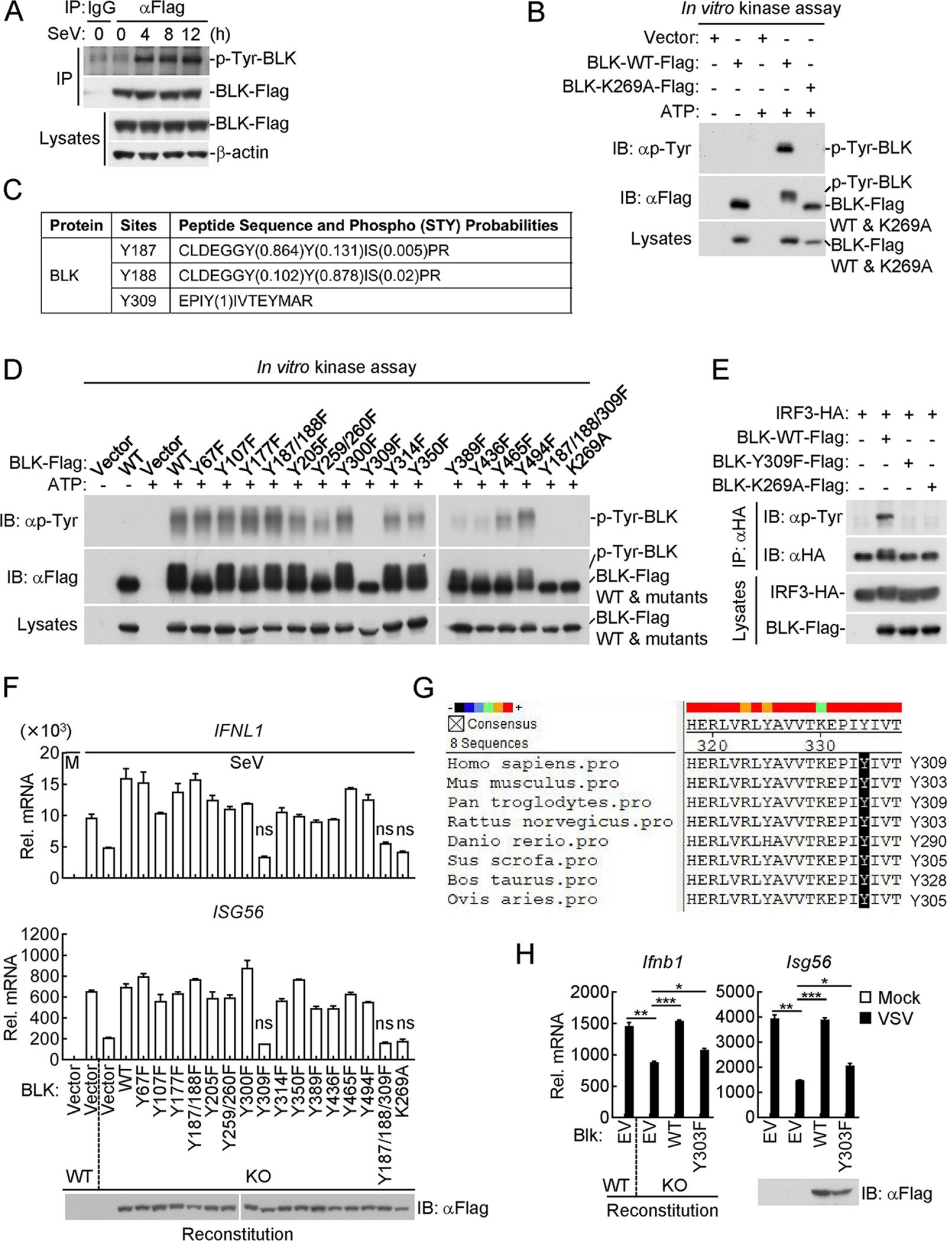
Autophosphorylation of BLK at Y309 is critical for its regulation of IRF3-dependent antiviral signaling. **(A)** BLK undergoes tyrosine phosphorylation upon SeV infection. U87MG cells stably expressing BLK-Flag (2 × 10^7^) were uninfected or infected with SeV (MOI, 1) for the indicated times. Coimmunoprecipitation and immunoblot analysis were performed with the indicated antibodies. **(B)** BLK catalyzes autophosphorylation in a kinase activity-dependent manner. Purified Flag-tagged BLK and BLK(K269A) coupled with Protein G sepharose beads were subjected to *in vitro* BLK autophosphorylation assays. The detailed procedures are shown in the Materials and Methods. “Vector” here means the control Protein G sepharose beads. **(C)** Identification of potential autophosphorylation sites of BLK by mass spectrometry. The *in vitro* phosphorylated BLK in Fig 7B was subjected to mass spectrometry. The list shows the phosphorylated peptide sequences and phosphorylation (STY) probabilities of BLK analyzed by mass spectrometry. **(D)** BLK catalyzes autophosphorylation mainly at Y309. Purified Flag-tagged BLK and its mutants coupled with Protein G sepharose beads were subjected to *in vitro* BLK autophosphorylation assays. **(E)** BLK(Y309F) fails to phosphorylate IRF3. HEK293 cells (2 × 10^6^) were transfected with the indicated plasmids for 24 h. Coimmunoprecipitation and immunoblot analysis were performed with the indicated antibodies. **(F)** Effects of BLK or its mutant recovery on SeV-induced transcription of downstream antiviral genes. BLK-deficient and control U87MG cells (2 × 10^5^) were transfected with the same amount of BLK or its mutants for 24 h. Cells were then uninfected or infected with SeV (MOI, 1) for 8 h before qPCR analysis. The lower blot shows the expression levels of BLK and its mutants as detected by anti-Flag antibody. **(G)** Sequence alignment of BLK from the indicated species. The sequences correspond to aa293-312 of human BLK. The conserved tyrosine residues are highlighted in black. **(H)** Effects of Blk or Blk(Y303F) recovery on VSV-induced transcription of downstream antiviral genes. Wild-type and Blk-deficient A20 cells were transduced with empty vector (EV), Blk or Blk(Y303F) plasmids by lentivirus-mediated gene transfer to establish the stable cell lines with blasticidin S (10 μg/mL) selection. The indicated cell lines (2 × 10^5^) were then uninfected or infected with VSV (MOI, 1) for 8 h before qPCR analysis. The lower blot shows the expression levels of Blk and Blk(Y303F) in the indicated cell lines as detected by anti-Flag antibody. Graphs show mean ± SD (*n* = 2 technical replicates in F and H) from one representative experiment. Data are representative of at least three independent experiments with similar results. *P < 0.05, **P < 0.01, ***P < 0.001, ns, not significant (unpaired, two-tailed Student’s *t*-test).

Previous studies have shown that several kinases catalyze their substrates accompanied with autophosphorylation, which is critical for self-activation and their regulation of cell survival, homeostasis, and infection prevention [55]. Unexpectedly, we noticed that wild-type BLK but not BLK(K269A) could also be phosphorylated (Fig 6D). We next performed the following experiments to verify whether BLK phosphorylation is mediated by itself. As shown in Fig 7A, coimmunoprecipitation analysis showed that SeV infection induced tyrosine phosphorylation of BLK. Further *in vitro* kinase assays showed that wild-type BLK but not BLK(K269A) indeed underwent tyrosine phosphorylation, and the shifted band with higher molecular weight represents the phosphorylated BLK (Fig 7B). To delineate which residues might be responsible for BLK autophosphorylation, the *in vitro* phosphorylated BLK in Fig 7B was subjected to mass spectrometry analysis, and the specific phosphorylation sites are listed in Fig 7C and S2 Table. Subsequently, we generated a series of BLK point mutants in which tyrosine residues were replaced with phenylalanine. As shown in Fig 7D, these mutants catalyzed the different levels of autophosphorylation *in vitro*. Most notably, the Y309F mutation resulted in an evident reduction or almost complete loss of BLK autophosphorylation (Fig 7D). Thus, these results suggest that BLK catalyzes autophosphorylation at Y309.

We next determined whether BLK autophosphorylation at Y309 is indispensable for its regulation of virus-induced innate antiviral signaling. As shown in Fig 7E, BLK(Y309F) failed to catalyze tyrosine phosphorylation of IRF3. Furthermore, SeV-induced transcription of downstream antiviral genes, including *IFNL1* and *ISG56*, was substantially inhibited in BLK(Y309F)-reconstituted cells in comparison to other mutant-reconstituted cells (Fig 7F). In these experiments, SeV-induced transcription levels of downstream genes in BLK-deficient and BLK(Y309F)-reconstituted cells were comparable (Fig 7F). These results indicate that the inability of BLK(Y309F) to phosphorylate IRF3 is attributed to a defect of BLK autophosphorylation activity, and BLK autophosphorylation further promotes tyrosine phosphorylation of IRF3, thereby augmenting IRF3-mediated production of downstream antiviral genes. Intriguingly, sequence alignment found that Y309 of BLK, corresponding to Y303 of murine Blk, is highly conserved among various species (Fig 7G). We wondered whether the murine Y303 residue exerts a similar function as the human Y309 residue in controlling IFN induction. Therefore, we reconstituted wild-type Blk or Blk(Y303F) mutant into Blk-deficient A20 cells by a pseudotyped lentivirus-mediated gene transfer method. As shown in Fig 7H, reconstitution of Blk-deficient cells with Blk but not Blk(Y303F) rescued VSV-induced transcription of downstream genes, including *Ifnb1* and *Isg56*. Collectively, these results suggest that autophosphorylation of BLK at Y309 or murine Blk at Y303 is required for their regulation of virus-triggered innate antiviral signaling.

### BLK facilitates sufficient phosphorylation and dimerization of IRF3 to enhance cellular antiviral response

**Fig 8:**
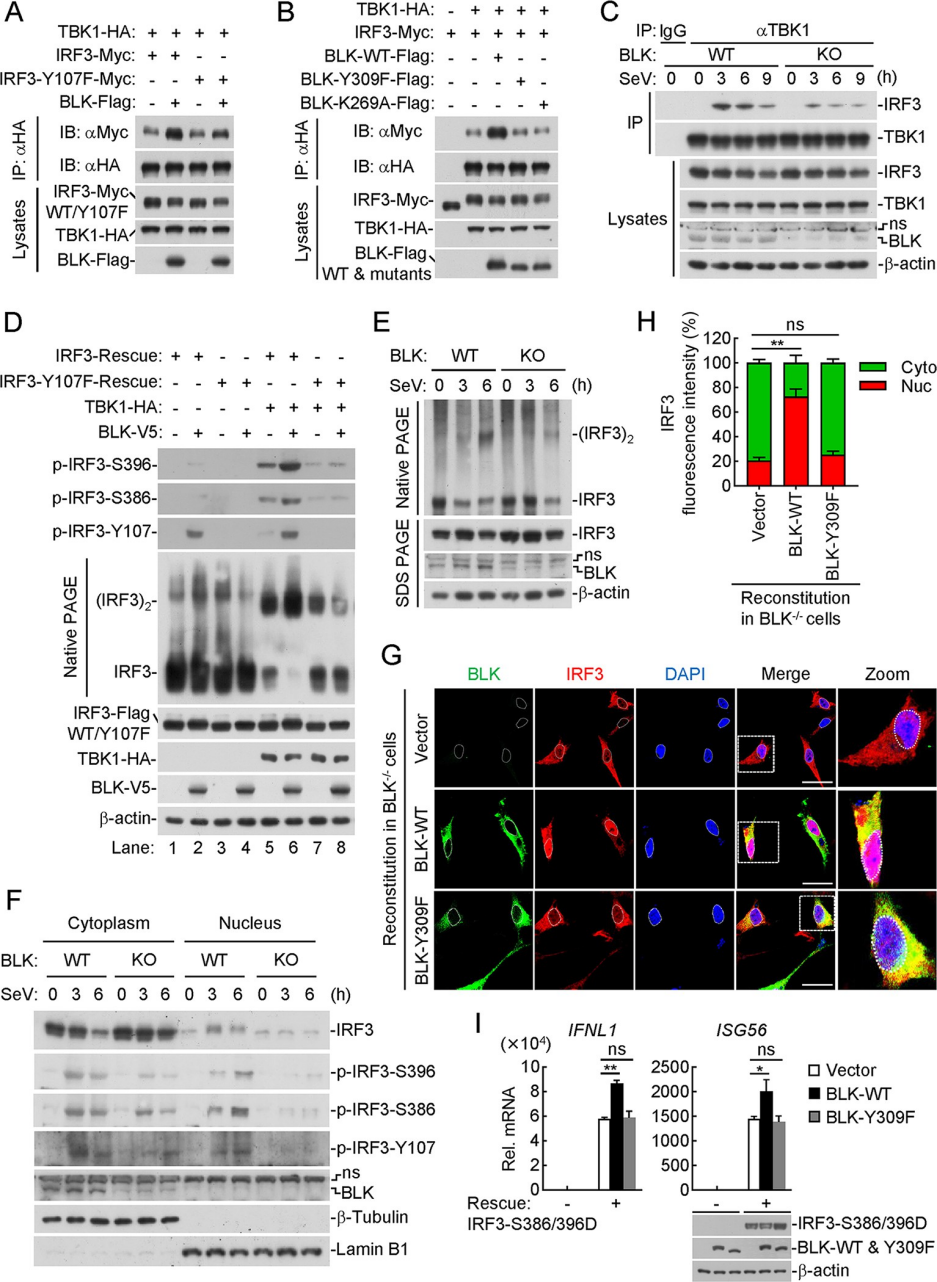
BLK promotes sufficient activation of IRF3 to facilitate cellular antiviral response. **(A and B)** BLK promotes the recruitment of IRF3 to TBK1 in a kinase activity-dependent manner. HEK293 cells (2 × 10^6^) were transfected with the indicated plasmids for 24 h. Coimmunoprecipitation and immunoblot analysis were performed with the indicated antibodies. **(C)** Effects of BLK deficiency on SeV-induced recruitment of IRF3 to TBK1. BLK-deficient and control U87MG cells (2 × 10^7^) were uninfected or infected with SeV (MOI, 1) for the indicated times before coimmunoprecipitation and immunoblot analysis. **(D)** Effects of BLK on TBK1-mediated phosphorylation and dimerization of IRF3. IRF3- or IRF3(Y107F)-reconstituted U87MG cells (5 × 10^5^) were co-transfected with the same amount of TBK1 or BLK plasmids for 24 h before native PAGE and SDS PAGE analyses with the indicated antibodies. **(E)** Effects of BLK deficiency on SeV-triggered dimerization of IRF3. BLK-deficient and control U87MG cells (1 × 10^6^) were uninfected or infected with SeV (MOI, 1) for the indicated times before native PAGE and SDS PAGE analyses. **(F)** Effects of BLK deficiency on SeV-triggered nuclear translocation of IRF3. BLK-deficient and control U87MG cells (1 × 10^6^) were uninfected or infected with SeV (MOI, 1) for the indicated times before the nuclear and cytoplasmic extraction assays. **(G and H)** Effects of BLK or BLK(Y309F) recovery on SeV-triggered nuclear translocation of IRF3. (G) BLK-deficient U87MG cells were transduced with BLK or BLK(Y309F) plasmids by lentivirus-mediated gene transfer to establish the stable cell lines with blasticidin S (10 μg/mL) selection. Subsequently, the indicated cell lines (1 × 10^5^) were infected with SeV (MOI, 1) for 4 h, then fixed with 4% paraformaldehyde and stained with anti-Flag and anti-IRF3 antibodies before confocal microscopy. Scale bars, 50 μm. (H) Quantitative analysis of IRF3 fluorescence intensity in the cytoplasm and nucleus in Fig 8G. Statistical analyses were based on colocalization images (covering dozens of cells) using ImageJ software. **(I)** Effects of BLK or BLK(Y309F) on IRF3(S386/396D)-induced transcription of downstream antiviral genes. IRF3-deficient U87MG cells (2 × 10^5^) were co-transfected with the indicated plasmids (IRF3(S386/396D) 0.5 μg, BLK 1.5 μg, BLK(Y309F) 1.5 μg) for 24 h before qPCR analysis. The lower blots show the expression levels of the indicated plasmids. Graphs show mean ± SD (*n* = 12 cells from three individual images in H, *n* = 2 technical replicates in I) from one representative experiment. Data are representative of at least three independent experiments with similar results. *P < 0.05, **P < 0.01, ns, not significant (unpaired, two-tailed Student’s *t*-test).

Previous studies have reported that IRF3 contains a C-terminal cluster of serine residues, in which the phosphorylation of IRF3 at S386 is mainly mediated by TBK1, and the phosphorylation of IRF3 at S396 is mediated by both TBK1 and IKKε [21–24]. Phosphorylation and dimerization are two hallmarks of IRF3 activation after viral infection [56–58]. We next detected whether BLK participates in the above IRF3 activation processes. Coimmunoprecipitation experiments indicated that BLK promoted the association of TBK1 with IRF3 but not IRF3(Y107F) (Fig 8A). In addition, BLK but not its mutants BLK(Y309F) or BLK(K269A) enhanced the interaction between TBK1 and IRF3 (Fig 8B). Endogenous coimmunoprecipitation experiments showed that BLK deficiency impaired SeV-induced recruitment of IRF3 to TBK1 (Fig 8C). These results suggest that BLK facilitates the recruitment of IRF3 to TBK1, which depends on the kinase activity of BLK.

We next examined whether BLK affects TBK1-mediated phosphorylation of IRF3 and its homodimerization. As shown in Fig 8D, IRF3-deficient cells were reconstituted with either IRF3 or IRF3(Y107F) together with TBK1 and BLK. TBK1 overexpression alone (without BLK) markedly enhanced IRF3 S386 and S396 phosphorylation as well as IRF3 dimerization when IRF3-deficient cells were reconstituted with wild-type IRF3 (Fig 8D, lane 5), which was further enhanced with BLK and TBK1 co-expression (Fig 8D, lane 6). Additionally, we noticed that TBK1 overexpression alone barely affected IRF3 Y107 phosphorylation (Fig 8D, lane 5), while BLK overexpression alone enhanced IRF3 Y107 phosphorylation (Fig 8D, lane 2). Notably, compared with BLK overexpression alone, TBK1 and BLK co-expression failed to enhance IRF3 Y107 phosphorylation (Fig 8D, lane 6). Moreover, TBK1-mediated IRF3 S386 and S396 phosphorylation as well as IRF3 dimerization were clearly inhibited in IRF3(Y107F)-reconstituted cells compared with IRF3-reconstituted cells (Fig 8D, lane 7), whereas BLK and TBK1 co-expression failed to enhance TBK1-mediated IRF3 S386 and S396 phosphorylation as well as IRF3 dimerization (Fig 8D, lane 8). Furthermore, BLK rather than BLK(Y309F) promoted SeV-induced IRF3 dimerization in IRF3-reconstituted cells (S5A Fig). As shown in Fig 8E, BLK deficiency impaired SeV-triggered endogenous IRF3 dimerization. Therefore, these data provide important evidence that IRF3 Y107 phosphorylation mediated by BLK facilitates TBK1 to further induce IRF3 S386 and S396 phosphorylation, thus achieving sufficient dimerization of IRF3.

We next detected whether BLK further affects IRF3 nuclear translocation. As shown in Fig 8F, in response to SeV infection, BLK deficiency promoted the cytoplasmic retention of IRF3 and thus impeded the nuclear translocation of IRF3. Moreover, BLK deficiency attenuated IRF3 phosphorylation at S386, S396, and Y107 sites in the cytoplasm, and the nuclear translocation of phosphorylated IRF3 at these sites was also substantially impaired. Confocal microscopy experiments indicated that SeV-induced nuclear translocation of IRF3 was enhanced by the reconstitution of BLK but not BLK(Y309F) into BLK-deficient cells (Fig 8G). Statistical analysis of IRF3 fluorescence intensity in the cytoplasm and nucleus also confirmed the above results (Fig 8H). Moreover, reconstitution of BLK-deficient cells with BLK but not BLK(Y309F) restored SeV-induced IRF3 Y107 phosphorylation (S5B Fig). Subsequently, we constructed the constitutively active IRF3, named IRF3(S386/396D), in which the serine residues 386 and 396 are all mutated to aspartic acid (D), and reconstituted it into IRF3-deficient cells. As shown in Fig 8I, overexpression of BLK but not BLK(Y309F) potentiated IRF3(S386/396D)-induced transcription of *IFNL1* and *ISG56* genes. Collectively, these results suggest that BLK is essential for virus-triggered phosphorylation, dimerization, and nuclear translocation of IRF3 and subsequent cellular antiviral responses.

## Discussion

Innate immune sensing of pathogens is necessary for the host to mount defense responses. Accumulating evidence suggests that IRF3-dependent signaling plays a pivotal role in host defense against microbial infection [2, 59, 60]. Decoding how these events are tightly regulated to promote efficient elimination of pathogenic microbes while avoiding excessive immune responses is quite important. In the present study, BLK was first identified as a positive regulator of IRF3-dependent innate antiviral signaling.

Several lines of evidence demonstrated that BLK deficiency markedly impaired RNA or DNA virus-triggered antiviral protein production *in vivo* and *in vitro*. In line with the reduced IFN production, BLK deficiency promoted VSV replication, as shown by the increased virus copies and VSV-GFP fluorescence. More importantly, we separated primary murine pDCs and further demonstrated that Blk deficiency indeed impaired RNA or DNA virus-induced cellular antiviral response. It is worth further investigating whether BLK has a conserved antiviral function in other types of murine cells. Moreover, we evaluated the role of BLK in host defense against viral infection using an *in vivo* xenograft tumor model and found that BLK deficiency enhanced SVA-induced oncolysis by inhibiting cellular antiviral response and promoting SVA proliferation. Altogether, multiple strategies consistently confirmed the critical role of BLK in innate antiviral response. Intriguingly, although TBK1 and IRF3 are the common effectors downstream of RIG-I-, MDA5-, and cGAS-mediated pathways, BLK deficiency specifically impaired IRF3 but not TBK1 phosphorylation. We therefore hypothesized that BLK might function downstream of TBK1 to regulate the production of IFNs in response to multiple viral stimuli.

In this study, IRF3 was identified as a potential factor in the BLK interactome by silver staining and mass spectrometry methods. Cellular and biochemical experiments further indicated that BLK directly interacted with IRF3. *In vitro* kinase assays confirmed that BLK phosphorylated IRF3 at Y107. Using a phosphorylated antibody that specifically detected IRF3 Y107, we found that BLK deficiency indeed impaired SeV-induced Y107 phosphorylation of endogenous IRF3. Thus, these findings uncovered a previously unidentified phosphorylation site of IRF3 during viral infection. More importantly, BLK-mediated phosphorylation of IRF3 was indispensable for its regulatory function in virus-triggered innate antiviral signaling. Intriguingly, IRF3 Y107 mutation could not completely block SeV-induced ISRE activation (Fig 6F), implying that BLK is necessary but not sufficient for IRF3 activation. Upon viral infection, IRF3 undergoes serial processes of phosphorylation. IRF3 is phosphorylated within its C-terminus on serine residues 385, 386, 396, 398, 402, and 405 as well as threonine 404, primarily by TBK1 [17, 61]. TBK1-mediated IRF3 phosphorylation has been studied and documented in detail. Furthermore, AKT3 enhances IRF3 S385 phosphorylation to facilitate IRF3 dimerization [18]. Nonreceptor tyrosine kinase c-Abl phosphorylates IRF3 at Y292 to potentiate TBK1-dependent IRF3 activation upon dsDNA or dsRNA stimulation [58]. It is largely unknown whether these kinases also affect IRF3 Y107 phosphorylation. Moreover, whether and how these kinases cooperatively manipulate IRF3 activity to achieve its full activation have yet to be defined and should be investigated in the future.

Previous studies have reported that autophosphorylation of some kinases is required for their catalytic activity and regulatory function [62–64]. Our results suggested that SeV infection triggered BLK autophosphorylation. Mass spectrometry and *in vitro* kinase analyses indicated that BLK catalyzed autophosphorylation mainly at Y309. Although mutations in individual tyrosine residues had slight effects on BLK autophosphorylation, it was indisputable that Y309 alteration dramatically or almost completely ablated BLK autophosphorylation. Moreover, BLK(Y309F) failed to phosphorylate IRF3, and BLK(Y309F) reconstitution in BLK-deficient cells failed to restore SeV-induced production of downstream antiviral genes. These results indicate that viral infection triggers BLK Y309 autophosphorylation, and activated BLK subsequently phosphorylates IRF3 at Y107 to elicit potent innate antiviral responses, highlighting the critical role of BLK autophosphorylation in the regulation of IRF3 full activation.

To date, various mediators have been reported to positively or negatively regulate type I IFN production by targeting IRF3 [57, 65]. However, the mechanistic details of IRF3 activation are not fully understood. Our results suggested that BLK directly phosphorylated IRF3 to promote the recruitment of IRF3 to TBK1, IRF3 S386/S396 phosphorylation, and IRF3 dimerization and nuclear translocation, whereas the mutant BLK(Y309F) failed to do so due to the loss of its autophosphorylation activity. TBK1-mediated phosphorylation of IRF3 at S386 and S396 is essential for IRF3 dimerization [22], but little is known about which factors contribute to this event. In this study, several approaches demonstrated that co-transfection of BLK and TBK1 enhanced TBK1-induced IRF3 S386/S396 phosphorylation but had no effect on BLK-mediated IRF3 Y107 phosphorylation, which indicated that the event of IRF3 Y107 phosphorylation occurred prior to IRF3 S386/S396 phosphorylation. Moreover, we also found that BLK-mediated IRF3 Y107 phosphorylation not only facilitated TBK1 to further induce IRF3 S386/S396 phosphorylation, but also directly promoted IRF3 dimerization. Therefore, as shown in Fig 8I, overexpression of BLK but not BLK(Y309F) catalyzed the phosphorylation of IRF3(S386/396D) at Y107, which further promoted the sufficient dimerization of IRF3(S386/396D) and transcription of *IFNL1* and *ISG56* genes. These regulatory mechanisms collaboratively promote the sufficient activation and dimerization of IRF3, thereby eliciting robust downstream antiviral response. Collectively, the most valuable finding from our study is that targeting BLK offers a protective function against viral infection by enhancing IRF3 activation and IFN production.

## Materials and methods

### Ethics statement

All animals were handled in strict accordance with good animal practices according to the Animal Ethics Procedures and Guidelines of the People’s Republic of China. Eight- to ten-week-old, age- and sex-matched mice were used in all the experiments. All mouse studies were approved by the Animal Ethics Committee of Lanzhou Veterinary Research Institute, Chinese Academy of Agricultural Sciences (approval number LVRIAEC-2023-035).

### Reagents, antibodies, cells and viruses

Dual-specific luciferase assay kit (Promega, E1980); RNAiso plus (Takara, 9109); SYBR Green (Bio-Rad, 172–5274); HiScript II Q RT SuperMix (Vazyme, R222-01); Polybrene (Millipore, TR-1003-G); DAPI (Sigma-Aldrich, D9542); poly(I:C)-HMW and poly(I:C)-LMW (Invivogen, tlrl-pic and tlrl-picw, respectively); Recombinant human IFN-β and IFN-γ (R&D Systems, 8499-IF and 285-IF, respectively); Protein G sepharose beads and Glutathione sepharose beads (GE Healthcare, 17-0618-05 and 17-0756-01, respectively); Silver Stain for Mass Spectrometry (Thermo Fisher, 24600); Mouse Plasmacytoid Dendritic Cell Isolation Kit (Miltenyi, 130-107-093); Matrigel (BD Biosciences, 356234); ELISA kits for human IFN-γ and IFN-λ1 (BioLegend, 430107 and 446307, respectively); ELISA kits for mouse IFN-α and IFN-β (PBL, 42120 and 42400, respectively); NE-PER Nuclear and Cytoplasmic Extraction Reagents (Thermo Fisher, 78833).

Mouse monoclonal antibodies against Flag (F3165) and β-actin (A2228) were purchased from Sigma; GFP (2955), BLK (3262), Myc (2276), β-Tubulin (2146), Lamin B1 (13435), phospho-IRF3 (Ser396) (4947), phospho-IRF3 (Ser386) (37829) and phospho-Tyrosine (P-Tyr-100) (9411) were purchased from Cell Signaling Technology; TBK1 (ab109735) and phospho-TBK1 (Ser172) (ab109272) were purchased from Abcam; HA (TA100012) was purchased from OriGene; IRF3 (sc-9082) was purchased from Santa Cruz Biotechnology; BLK (A7427) and V5 (AE017) were purchased from ABclonal Technology; A purified rabbit polyclonal antibody phospho-IRF3 (Y107) was raised specifically against a modified peptide DPHDPHKIY(P)EFV; The secondary antibody goat anti-mouse (31430) and goat anti-rabbit (31460) IgG conjugated to HRP were purchased from Pierce; Goat anti-Rabbit IgG (H+L) Highly Cross-Adsorbed Secondary Antibody (A-11034), Goat anti-Mouse IgG (H+L) Cross-Adsorbed Secondary Antibody (A-11001), Goat anti-Mouse IgG (H+L) Cross-Adsorbed Secondary Antibody (A-21422) and Goat anti-Rabbit IgG (H+L) Highly Cross-Adsorbed Secondary Antibody (A-21429) were purchased from Thermo Fisher.

HEK293 and Vero cells were purchased from ATCC; U87MG, Jurkat, Raji and A20 cells were kindly provided by Procell Life Science & Technology Co., Ltd; IBRS-2 cells were previously described [66]; SeV (Cantell strain), VSV (Indiana strain), EMCV (BJC3 strain) and HSV-1 (KOS strain) were previously described [10, 67, 68]; HCMV (AD169) was provided by Dr. Min-Hua Luo (Wuhan Institute of Virology, CAS); SVA (CH-FJ-2017 strain) was isolated and stored in our laboratory previously [69].

### Constructs

Overexpression plasmids were constructed into pCMV14 or pRK vectors by standard molecular cloning methods. The point mutation plasmids were constructed by site-directed mutagenesis methods. BLK or IRF3 was constructed into pGEX-6P-1 with a GST tag in its N-terminus. IRF3 was constructed into pET-30c (+) with a His tag in its N-terminus. BLK was constructed into pRK with a GST tag in its N-terminus. BLK or its mutants were constructed into pLX304 with a V5 tag in its C-terminus. BLK, IRF3 or their mutants were constructed into pMSCV with a Flag tag in its C-terminus. All gRNAs were constructed into lentiCRISPR v2. IFN-β, ISRE, STAT1/2 and IRF1 reporter plasmids were previously described [8, 10, 13].

### Transfection and reporter assays

The cells were transfected by standard calcium phosphate precipitation or Lipofectamine 2000. To normalize the transfection efficiency, pRL-TK (*Renilla* luciferase) reporter plasmid (10 ng) was added to each transfection. To ensure that each transfection received the same amount of total plasmid DNA, the empty control plasmid was added to each transfection. Twenty-four hours after transfection, cells were treated or untreated with the indicated stimuli before luciferase assays were performed using a dual-specific luciferase assay kit. Firefly luciferase activities were normalized on the basis of *Renilla* luciferase activities.

### RNA extraction and qPCR

Total RNA was isolated from cells using RNAiso plus reagent. After reverse-transcription with HiScript II Q RT SuperMix, the cDNA was diluted 50-fold and subjected to qPCR analysis to measure mRNA levels of the tested genes. Data shown were the relative abundance of the indicated mRNAs normalized to that of *GAPDH*. Gene-specific primer sequences were as follows:

*GAPDH*: GACAAGCTTCCCGTTCTCAG (forward) and GAGTCAACGGATTTGGTCGT (reverse),

*IFNB1*: TTGTTGAGAACCTCCTGGCT (forward) and

TGACTATGGTCCAGGCACAG (reverse),

*IFNL1*: AACTGGGAAGGGCTGCCACATT (forward) and

GGAAGACAGGAGAGCTGCAACT (reverse),

*ISG56*: TCATCAGGTCAAGGATAGTC (forward) and

CCACACTGTATTTGGTGTCTAGG (reverse),

*ISG54*: GGAGCAGATTCTGAGGCTTTGC (forward) and

GGATGAGGCTTCCAGACTCCAA (reverse),

*RIG-I*: ACGCAGCCTGCAAGCCTTCC (forward) and

TGTGGCAGCCTCCATTGGGC (reverse),

*IRF7*: CCCCCATCTTCGACTTCAGA (forward) and

CAGGACCAGGCTCTTCTCCTT (reverse),

*IRF1*: GAGGAGGTGAAAGACCAGAGCA (forward) and

TAGCATCTCGGCTGGACTTCGA (reverse),

*GBP1*: TAGCAGACTTCTGTTCCTACATCT (forward) and

CCACTGCTGATGGCATTGACGT (reverse),

*SOCS1*: TTCGCCCTTAGCGTGAAGATGG (forward) and

TAGTGCTCCAGCAGCTCGAAGA (reverse),

*BLK*: AGGAAAAGCCGATCAAAGAGAAG (forward) and

CCACCACGAAATGCTTGTCT (reverse),

*Gapdh*: ACGGCCGCATCTTCTTGTGCA (forward) and

ACGGCCAAATCCGTTCACACC (reverse),

*Ifnb1*: TCCTGCTGTGCTTCTCCACCACA (forward) and

AAGTCCGCCCTGTAGGTGAGGTT (reverse),

*Ifnl2*: CCAGTGGAAGCAAAGGATTGCC (forward) and

TCAGGTCCTTCTCAAGCAGCCT (reverse),

*Isg56*: ACAGCAACCATGGGAGAGAATGCTG (forward) and

ACGTAGGCCAGGAGGTTGTGCAT (reverse),

*Blk*: CAGTTTGGCGAAGTCTGGATGG (forward) and

AGACGAACCAGCCTCTCATGCT (reverse),

*SVA-5’UTR*: AACCGGCTGTGTTTGCTAGAG (forward) and

GAACTCGCAGACCACACCAA (reverse).

### Coimmunoprecipitation and immunoblot analysis

Cells were lysed in l mL NP-40 lysis buffer (20 mM Tris-HCl, pH 7.5, 150 mM NaCl, 1 mM EDTA, 1% NP-40) supplemented with protease and phosphatase inhibitors. The lysates were centrifuged at 12,000 rpm for 15 min at 4°C. The supernatant was divided into two equal portions and incubated with 30 μL Protein G sepharose beads and the indicated antibody or control IgG (1 μg) at 4°C for 3 h. Beads were then washed three times with lysis buffer containing 0.5 M NaCl. The precipitates were resuspended in 2 × SDS loading buffer (50 μL), boiled for 15 min, and further analyzed by standard immunoblot procedures.

### Mice

*Blk*^-/-^ mice with the C57BL/6J background were generated utilizing the CRISPR/Cas9 method. The strategy for construction of the targeting vector was illustrated in S3A Fig. Exon 3–9 and part of intron of *Blk* gene were targeted by four specific gRNAs, which led to the deletion of exon 3–9 (~7.8 kb) of *Blk* coding sequence. Genotyping was performed by PCR with the following primers: P1: 5’-GGCAACTCAGAGAGGAAAGG-3’ (forward), P2: 5’-TTGAAAACTGCAGCCAGGAG-3’ (reverse), and P3: 5’-CTAGAGGACACTGGAGAGCC-3’ (reverse). Amplification of the WT allele produced a 545 bp fragment, and amplification of the disrupted allele produced a 323 bp fragment.

### Isolation of primary murine pDCs

Spleens were obtained from 2-month-old C57BL/6J background mice. A suspension of single splenocytes was obtained by passage of the spleens through a 40-μm-mesh-size cell strainer after lysis of red blood cells with ACK lysis buffer. Splenic pDCs were purified by negative magnetic selection using the Mouse Plasmacytoid Dendritic Cell Isolation Kit. pDCs were then infected with VSV or HSV-1 for the indicated times before qPCR and immunoblot analysis.

### VSV infection of mice

Age- and sex-matched *Blk*^+/+^ and *Blk*^-/-^ mice were i.p. infected with VSV. The survival of the infected mice was monitored for 16 days. The blood was collected at 6 h post VSV infection for measurement of IFN-α and IFN-β by ELISA.

### *In vivo* SVA efficacy

Athymic female mice (BALB/c-nu), aged 6–8 weeks, were injected subcutaneously in the flank with wild-type or BLK-KO U87MG cells (5 × 10^6^ cells per mouse) in 10% phenol red-free Matrigel. Once tumors reached volumes of approximately 100 mm^3^ (~ 7 days), mice within each cohort were randomly distributed and challenged with SVA (1 × 10^8^ PFU per mouse) or an equivalent volume of PBS, pH 7.4, via intravenous injection.

Twenty-four hours after SVA challenge, tumors (n = 7 for each group) were excised, and the same mass of tumor tissue was homogenized for 10 s in PBS. The suspensions were centrifuged at 1,620 g for 10 min, and the supernatants were collected for ELISA measurement of the indicated cytokine levels according to the manufacturer’s instructions.

Three days after SVA challenge, tumors (n = 4 for each group) were excised, and the same mass of tumor tissue was homogenized for 10 s in PBS. The suspensions were centrifuged at 1,620 g for 10 min, and the supernatants were subjected to viral plaque and qPCR assays, respectively.

For *in vivo* antitumor efficacy studies, tumors (n = 3 for each group) were measured in two dimensions with Vernier calipers every 2 days, and tumor volumes were calculated using the formula *V* = (*L* × *W*^2^)/2, where *L* is the length or diameter and *W* is the width. Each data point represents the average tumor volume with error bars representing the SD and was plotted using GraphPad Prism 6 software. At the end of the study, mice were euthanized by CO_2_ asphyxiation.

### Viral plaque assays

Eight week-old mice were infected with VSV for 4 days, the spleens of mice were weighted and homogenized for 10 s in PBS. After homogenization, the spleen suspensions were centrifuged at 1,620 g for 10 min, and the supernatants were collected for plaque assays. Vero or IBRS-2 cells were seeded in 24-well plates, and the cells were infected with serial dilutions of the spleen or tumor tissue supernatants at 37°C for 2 h, overlaid with 2% methylcellulose and further incubated for 36–48 h. The overlay was then removed, and the cells were fixed with 4% paraformaldehyde for 30 min and stained with 1% crystal violet for 30 min before plaque counting.

### Silver staining and mass spectrometry analyses

HEK293 cells (2 × 10^7^) were transfected with GST-BLK or GST plasmids for 24 h. Cells were then infected with SeV (MOI, 1) for 4 h before treatment with NP-40 lysis buffer containing protease and phosphatase inhibitors. GST-BLK-associated factors were immunoprecipitated from lysate supernatant using glutathione sepharose beads. Immunoprecipitated proteins were detected by immunoblot followed by silver staining. The gel bands were separated and further analyzed by mass spectrometry.

### Recombinant protein purification and *in vitro* GST pull-down assays

Recombinant GST-BLK, GST-IRF3 and His-IRF3 proteins expressed in *E*. *coli* Rosetta strain were purified with glutathione sepharose or Ni-NTA resin by the AKTA protein purification system. TBK1-Flag and IRF3-Flag proteins expressed in HEK293 cells were purified with Protein G sepharose beads and eluted from beads with 3×Flag peptide (1 mg/mL). Then, His-IRF3, TBK1-Flag, or IRF3-Flag protein was added to the purified recombinant GST-BLK protein coupled to glutathione sepharose beads and incubated for 3 h at 4°C. Subsequently, the beads were washed and boiled. The eluates/inputs were fractionated by SDS-PAGE and detected by Coomassie staining or immunoblot analysis.

### *In vitro* tyrosine phosphorylation assays

Recombinant GST-IRF3 protein expressed in *E*. *coli* Rosetta strain was purified with glutathione sepharose and eluted from beads with elution buffer (50 mM Tris-HCl, pH 8.0, 10 mM reduced glutathione). For *in vitro* kinase assays, vector, Flag-tagged BLK and its mutant plasmids were individually transfected into HEK293 cells for 24 h. These proteins were then purified with Protein G sepharose beads and co-incubated with purified GST-IRF3 (50 μg) in an equal volume of 2 × reaction buffer (100 mM Tris-HCl, 20 mM MgCl_2_, 1 mM Na_3_VO_4_, 4 mM DTT, pH 7.2), and 1 mM ATP was added prior to incubation for 1 h at 30°C. The reaction was stopped by adding 1/5 volume of 6 × SDS loading buffer and boiled for 15 min at 95°C before immunoblot analysis. For BLK autophosphorylation assays, vector, Flag-tagged BLK and its mutants plasmids were individually transfected into HEK293 cells for 24 h. These proteins were then purified with Protein G sepharose beads and incubated with 50 μL of 1 × reaction buffer (50 mM Tris-HCl, 10 mM MgCl_2_, 0.5 mM Na_3_VO_4_, 2 mM DTT, pH 7.2), and 1 mM ATP was added prior to incubation for 1 h at 30°C. The reaction was stopped by adding 1/5 volume of 6 × SDS loading buffer and boiled for 15 min at 95°C before immunoblot analysis.

### CRISPR/Cas9 knockout

Double-stranded oligonucleotides corresponding to the target sequences were cloned into the lentiCRISPR v2 vector. The constructed plasmid (10 μg) was then co-transfected with two packaging plasmids (LH1 (7.5 μg) and LH2 (5 μg)) into HEK293 cells. The culture medium was replaced with fresh medium without antibiotics 12 h after transfection. After an additional 36 h, the medium containing lentiviral particles was filtered (0.22 μm filter, Millipore) and used to infect Jurkat, Raji, U87MG or A20 cells in the presence of polybrene (8 μg/mL). The infected cells were selected with puromycin (1 μg/mL) or blasticidin S (10 μg/mL) for at least 6 days before additional experiments were performed. The corresponding gRNA oligonucleotide sequences were as follows:

human BLK gRNA #1: AAGTAGCAAAAAGCCGGACA,

human BLK gRNA #2: CCGATCAAAGAGAAGGACAA,

human IRF3 gRNA: GCTGGTGTCGCAGCTGGACC,

mouse Blk gRNA #1: GGTAAACACCCCGAAGTGGA,

mouse Blk gRNA #2: CCTGGGTGCGGATCTTCACG,

mouse Irf3 gRNA: GAACGAGGTTCAGGATCCCG.

### Establishment of stable cell lines

The plasmids pMSCV-BLK or pMSCV-IRF3 (10 μg) and two packaging vectors (Gag-pol (10 μg) and VSV-G (3 μg)) were cotransfected into HEK293 cells. The detailed procedure is similar to the CRISPR/Cas9 knockout method described above.

### RNA interference experiments

The siRNA duplexes targeting BLK were chemically synthesized by GenePharma. The siRNA duplexes were transfected into cells with PepMute siRNA transfection reagent (SignaGen Laboratories) according to the manufacturer’s instructions. The following sequences of human *BLK* mRNA were targeted:

BLK-RNAi #1: AAGCGACTGTCATCAAGTA,

BLK-RNAi #2: AGGTGGTTCTTTAGATCAC,

Negative control (NC): TTCTCCGAACGTGTCACGT.

### Flow cytometry for quantitation of VSV-GFP-infected cells

VSV-GFP-infected wild-type and Blk-deficient pDCs were collected and washed with PBS. Cells were then resuspended in 200 μL FACS buffer (PBS containing 1% fetal calf serum and 10 mM EDTA) and filtered with a 300-mesh strainer before flow cytometry using a BD Fortessa flow cytometer. Data were analyzed using FlowJo software (Tree Star).

### Confocal microscopy

Cells were infected with SeV for the indicated times, then fixed with 4% paraformaldehyde for 10 min and permeabilized with 0.1% Triton X-100 in PBS for 10 min at 4°C. Cells were blocked with 1% BSA in PBS and stained with the indicated primary and secondary antibodies for 2 h. The nuclei were stained with DAPI. Cells were observed with Nikon confocal microscope under a 60× oil objective. Quantitative analysis of colocalization images was performed using the open source Fiji (ImageJ) software.

### Native PAGE

Control or virus-infected cells were collected with ice-cold PBS and lysed with lysis buffer (50 mM Tris-HCl, 150 mM NaCl, 1 mM EDTA, 1% NP-40, pH 7.4) containing protease inhibitors. The lysates were centrifuged at 12,000 rpm for 15 min. The supernatants were then subjected to native PAGE on an 8% gel without SDS. The gels were pre-run with running buffer (25 mM Tris-HCl, 192 mM Glycine, pH 8.4, with or without 0.2% deoxycholate in the cathode and anode buffer, respectively) at 75 V for 30 min. Subsequently, the samples were electrophoresed at 75 V for 3 h at a cold temperature and further transferred onto NC membranes for immunoblot analysis.

### Preparation of cytoplasmic and nuclear proteins

The assays were performed with a NE-PER nuclear and cytoplasmic extraction reagent kit according to the manufacturer’s instructions. Briefly, SeV- or HSV-infected cells were collected with ice-cold PBS and lysed by blowing 30 times with a 1 mL syringe in 500 μL membrane lysis buffer (10 mM HEPES, 10 mM KCl, 0.1 mM EDTA, 0.4% NP-40, pH 7.9) containing protease inhibitors. The homogenate was centrifuged at 500 g for 10 min. The supernatant was saved as cytosol, and the pellet was saved as crude nuclei. The crude nuclei were washed twice with 500 μL membrane lysis buffer and resuspended in 20–50 μL of extract buffer (20 mM Hepes, 0.4 M NaCl, 1 mM EDTA, pH 7.9) and shaken vigorously every 30 s for 15 min, followed by centrifugation at 12,000 rpm for 10 min. The supernatants containing nuclear or cytoplasmic proteins were subjected to immunoblot analysis.

### Statistical analysis

Unpaired, two-tailed Student’s *t* test was used for statistical analysis, and the F test was performed to confirm that two populations had the same variances. The Shapiro-Wilk normality test was performed to confirm the normal distribution of all datasets. For the mice survival study, Kaplan-Meier survival curves were generated and analyzed by the log-rank test. Statistical differences were evaluated using GraphPad Prism 8 software. *P* < 0.05 was considered significant.

## Supporting information

S1 Fig BLK specifically enhances IRF3-dependent antiviral signaling, related to Fig 1. **(A)** Effects of BLK on IFN-β-induced activation of STAT1/2. U87MG cells (1 × 10^5^) were co-transfected with STAT1/2 reporter (0.02 μg) and increased amounts of BLK expression plasmids (0.01, 0.05 μg) for 24 h. Cells were then left untreated or treated with IFN-β (100 ng/mL) for 10 h before luciferase assays. **(B)** Effects of BLK on IFN-γ-induced activation of IRF1. U87MG cells (1 × 10^5^) were co-transfected with IRF1 reporter (0.05 μg), pRL-TK (*Renilla* luciferase) reporter (0.01 μg) and increased amounts of BLK expression plasmids (0.01, 0.05 μg) for 24 h. Cells were then left untreated or treated with IFN-γ (100 ng/mL) for 10 h before luciferase assays. **(C and D)** Effects of BLK on SeV- or poly(I:C)-HMW-induced transcription of downstream antiviral genes. Jurkat (C) or U87MG (D) cells were transduced with vector (Vec) or BLK expression plasmids by lentivirus-mediated gene transfer to establish the stable cell lines. Cells (2 × 10^5^) were then infected with SeV (MOI, 1) for 8 h or transfected with poly(I:C)-HMW (1 μg) for 4 h before qPCR analysis. **(E)** Effects of BLK on IFN-β- or IFN-γ-induced transcription of downstream genes. U87MG cells stably expressing BLK and control plasmids (2 × 10^5^) were left untreated or treated with IFN-β (100 ng/mL) or IFN-γ (100 ng/mL) for 2 h before qPCR analysis. **(F-H)** The expression of BLK in different cell lines. Jurkat (F), Raji (G) or U87MG (H) cells stably expressing BLK and control plasmids (2 × 10^5^) were subjected to immunoblot analysis with anti-Flag antibody. Graphs show mean ± SD (*n* = 2 biological replicates in A and B, *n* = 2 technical replicates in C-E) from one representative experiment. Data are representative of at least three independent experiments with similar results. *P < 0.05, **P < 0.01 (unpaired, two-tailed Student’s *t*-test).
(TIF)

S2 Fig BLK deficiency attenuates virus-triggered innate immune response, related to Fig 1. **(A, B, and D)** Effects of BLK deficiency on dsRNA- or virus-induced transcription of downstream antiviral genes. BLK-deficient and control Jurkat (A), Raji (B), or U87MG (D) cells (2 × 10^5^) were infected with SeV (MOI, 1), HSV-1 (MOI, 1), HCMV (MOI, 5), or SVA (MOI, 1) for the indicated times or transfected with poly(I:C)-LMW (1 μg) or poly(I:C)-HMW (1 μg) for 4 h before qPCR analysis. **(C)** Effects of BLK knockdown on dsRNA- or SeV-induced transcription of downstream antiviral genes. U87MG cells (4 × 10^5^) were transfected with siRNA targeting human BLK for 48 h. Cells were then infected with SeV (MOI, 1) for the indicated times or transfected with poly(I:C)-LMW (1 μg) or poly(I:C)-HMW (1 μg) for 4 h before qPCR analysis. **(E)** Effects of BLK knockdown on EMCV-induced phosphorylation of TBK1 and IRF3. U87MG cells (4 × 10^5^) were transfected with siRNA targeting human BLK for 48 h. Cells were then infected with EMCV (MOI, 1) for the indicated times before immunoblot analysis. Graphs show mean ± SD (*n* = 2 technical replicates in A-D) from one representative experiment. Data are representative of at least three independent experiments with similar results. *P < 0.05, **P < 0.01, ***P < 0.001 (unpaired, two-tailed Student’s *t*-test).
(TIF)

S3 Fig Genotyping of Blk^-/-^ mice, related to Fig 2. **(A)** *Blk* gene targeting strategy. The exon 3–9 and part of intron of *Blk* gene were deleted by CRISPR/Cas9 method. **(B)** Genotyping of *Blk*^-/-^ mice. PCR analysis of genomic DNA to identify wild-type, heterogenous and *Blk*^-/-^ mice.
(TIF)

S4 Fig BLK mediates tyrosine phosphorylation of IRF3, related to Fig 6. **(A)** The expression levels of BLK and BLK(K269A). U87MG cells (2 × 10^5^) were transfected with BLK or BLK(K269A) plasmids for 24 h. Cells were then harvested for immunoblot analysis. **(B)** BLK mediates tyrosine phosphorylation of IRF3. HEK293 cells (2 × 10^6^) were transfected with the indicated plasmids for 24 h. Coimmunoprecipitation and immunoblot analysis were performed with the indicated antibodies. **(C)** Quantitative analysis of IRF3 fluorescence intensity in the cytoplasm and nucleus in Fig 6G. Statistical analyses were based on images (covering dozens of cells) using ImageJ software. **(D)** Sequence alignment of IRF3 from the indicated species. The sequences correspond to aa95-113 of human IRF3. The conserved tyrosine residues are highlighted in black. **(E)** Effects of Irf3 or Irf3(Y107F) recovery on VSV-induced transcription of downstream antiviral genes. A20 cells were transduced with gRNA plasmids targeting murine Irf3 by the CRISPR/Cas9 method to establish the stable cell lines with puromycin (1 μg/mL) selection. Wild-type and Irf3-deficient A20 cells were transduced with empty vector (EV), Irf3, or Irf3(Y107F) plasmids by lentivirus-mediated gene transfer to establish the stable cell lines with blasticidin S (10 μg/mL) selection. The indicated cell lines (2 × 10^5^) were then uninfected or infected with VSV (MOI, 1) for 8 h before qPCR analysis. The lower blots show the expression levels of Irf3 and Irf3(Y107F) in the indicated cell lines as detected by anti-Flag or anti-Irf3 antibodies, respectively. Graphs show mean ± SD (*n* = 12 cells from three individual images in C, *n* = 2 technical replicates in E) from one representative experiment. Data are representative of at least three independent experiments with similar results. *P < 0.05, **P < 0.01, ***P < 0.001 (unpaired, two-tailed Student’s *t*-test).
(TIF)

S5 Fig BLK deficiency impairs virus-induced IRF3 dimerization and nuclear translocation, related to Fig 8. **(A)** Effects of BLK or BLK(Y309F) on SeV-triggered IRF3 dimerization. IRF3-reconstituted U87MG cells (5 × 10^5^) were transfected with the same amount of BLK or BLK(Y309F) plasmids for 24 h. Cells were then uninfected or infected with SeV (MOI, 1) for 6 h before native PAGE and SDS PAGE analyses. **(B)** Effects of BLK or BLK(Y309F) recovery on SeV-triggered IRF3 Y107 phosphorylation. BLK- or BLK(Y309F)-reconstituted U87MG cells (1 × 10^5^) were infected with SeV (MOI, 1) for 6 h, then fixed with 4% paraformaldehyde and stained with anti-Flag and anti-phospho-IRF3 (Y107) antibodies before confocal microscopy. Scale bars, 50 μm. Data are representative of at least three independent experiments with similar results.
(TIF)

S1 Data Excel spreadsheet containing, in separate sheets, the underlying numerical data for Figs 1A–1E, 2A, 2D, 2G, 3A–3D, 4B–4E, 6A, 6F, 7F, 7H, 8H, 8I, S1A–S1E, S2A–S2D, S4C and S4E. (XLSX)

S1 Table List of BLK interacting proteins identified by mass spectrometric analysis. (XLSX)

S2 Table List of the potential autophosphorylation sites of BLK. (XLSX)
