# Supplementary figures and images for: Tyrosine phosphorylation of IRF3 by BLK facilitates its sufficient activation and innate antiviral response
Source: PLoS Pathog. 2023 Oct 23;19(10):e1011742. doi: 10.1371/journal.ppat.1011742 (PMC10621992; doi:10.1371/journal.ppat.1011742)

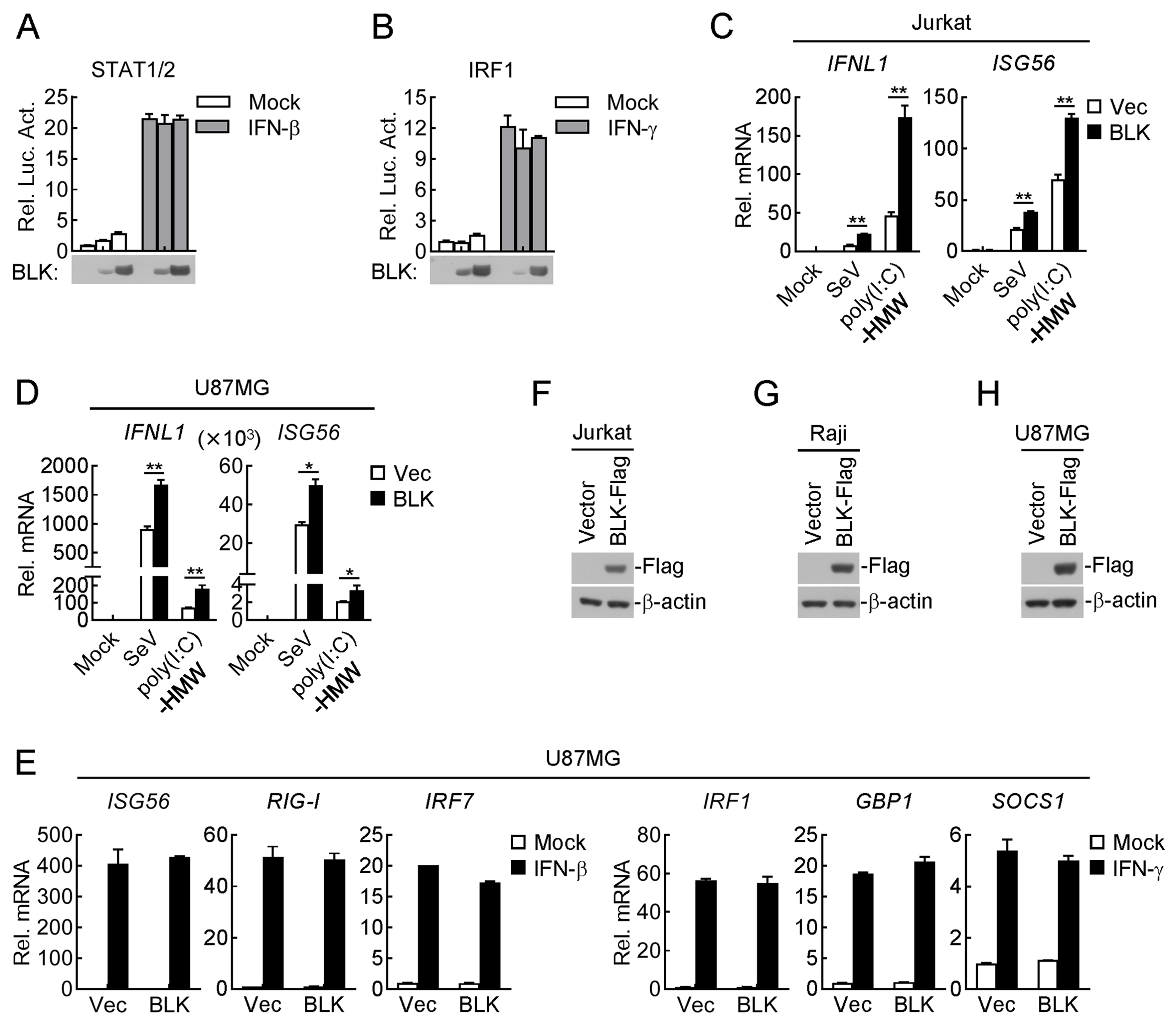

Supplement: S1 Fig — (A) Effects of BLK on IFN-β-induced activation of STAT1/2. U87MG cells (1 × 10^5^) were co-transfected with STAT1/2 reporter (0.02 μg) and increased amounts of BLK expression plasmids (0.01, 0.05 μg) for 24 h. Cells were then left untreated or treated with IFN-β (100 ng/mL) for 10 h before luciferase assays. (B) Effects of BLK on IFN-γ-induced activation of IRF1. U87MG cells (1 × 10^5^) were co-transfected with IRF1 reporter (0.05 μg), pRL-TK (Renilla luciferase) reporter (0.01 μg) and increased amounts of BLK expression plasmids (0.01, 0.05 μg) for 24 h. Cells were then left untreated or treated with IFN-γ (100 ng/mL) for 10 h before luciferase assays. (C and D) Effects of BLK on SeV- or poly(I:C)-HMW-induced transcription of downstream antiviral genes. Jurkat (C) or U87MG (D) cells were transduced with vector (Vec) or BLK expression plasmids by lentivirus-mediated gene transfer to establish the stable cell lines. Cells (2 × 10^5^) were then infected with SeV (MOI, 1) for 8 h or transfected with poly(I:C)-HMW (1 μg) for 4 h before qPCR analysis. (E) Effects of BLK on IFN-β- or IFN-γ-induced transcription of downstream genes. U87MG cells stably expressing BLK and control plasmids (2 × 10^5^) were left untreated or treated with IFN-β (100 ng/mL) or IFN-γ (100 ng/mL) for 2 h before qPCR analysis. (F-H) The expression of BLK in different cell lines. Jurkat (F), Raji (G) or U87MG (H) cells stably expressing BLK and control plasmids (2 × 10^5^) were subjected to immunoblot analysis with anti-Flag antibody. Graphs show mean ± SD (n = 2 biological replicates in A and B, n = 2 technical replicates in C-E) from one representative experiment. Data are representative of at least three independent experiments with similar results. *P < 0.05, **P < 0.01 (unpaired, two-tailed Student’s t-test). (TIF) [file ppat.1011742.s001.tif]

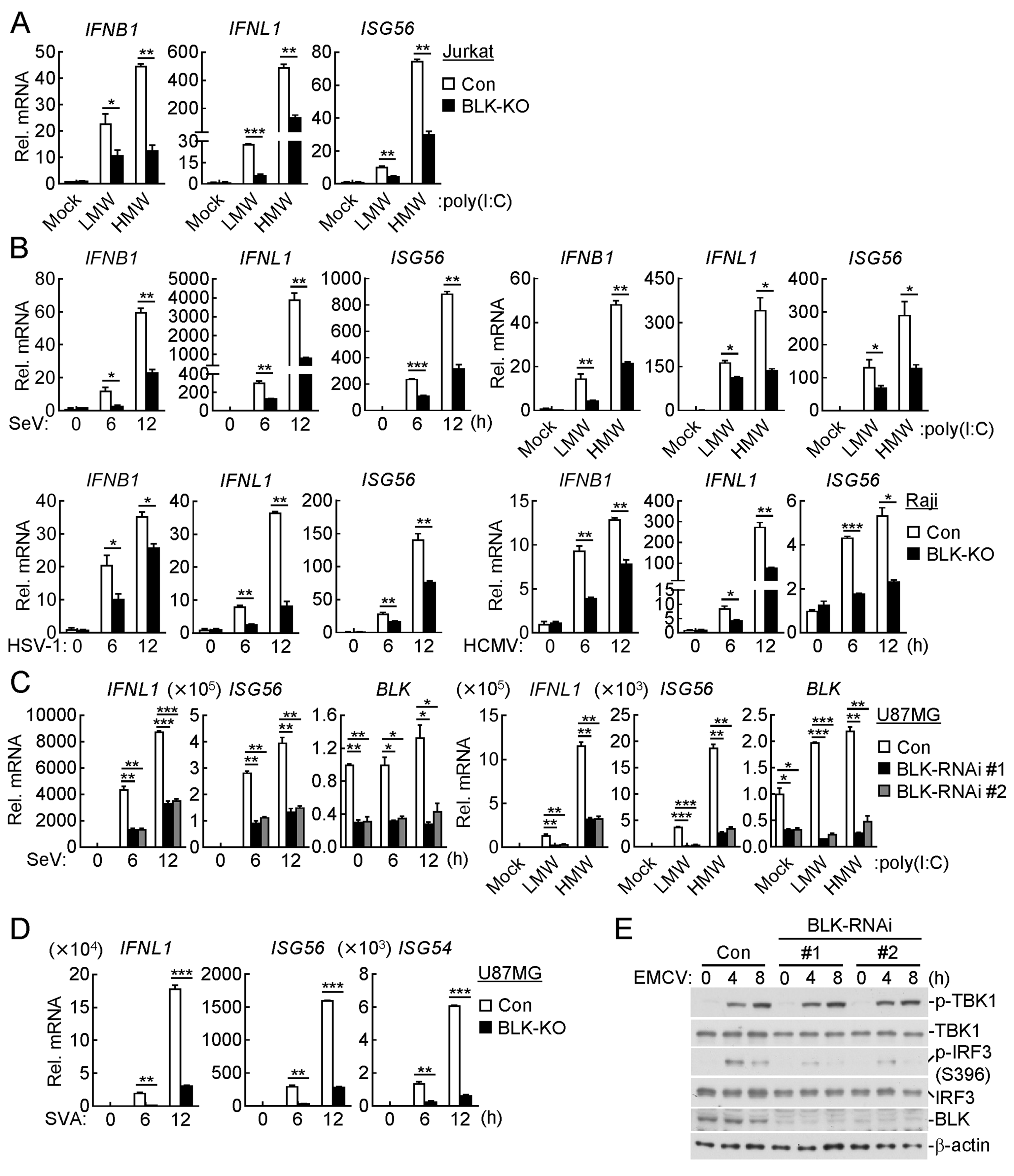

Supplement: S2 Fig — (A, B, and D) Effects of BLK deficiency on dsRNA- or virus-induced transcription of downstream antiviral genes. BLK-deficient and control Jurkat (A), Raji (B), or U87MG (D) cells (2 × 10^5^) were infected with SeV (MOI, 1), HSV-1 (MOI, 1), HCMV (MOI, 5), or SVA (MOI, 1) for the indicated times or transfected with poly(I:C)-LMW (1 μg) or poly(I:C)-HMW (1 μg) for 4 h before qPCR analysis. (C) Effects of BLK knockdown on dsRNA- or SeV-induced transcription of downstream antiviral genes. U87MG cells (4 × 10^5^) were transfected with siRNA targeting human BLK for 48 h. Cells were then infected with SeV (MOI, 1) for the indicated times or transfected with poly(I:C)-LMW (1 μg) or poly(I:C)-HMW (1 μg) for 4 h before qPCR analysis. (E) Effects of BLK knockdown on EMCV-induced phosphorylation of TBK1 and IRF3. U87MG cells (4 × 10^5^) were transfected with siRNA targeting human BLK for 48 h. Cells were then infected with EMCV (MOI, 1) for the indicated times before immunoblot analysis. Graphs show mean ± SD (n = 2 technical replicates in A-D) from one representative experiment. Data are representative of at least three independent experiments with similar results. *P < 0.05, **P < 0.01, ***P < 0.001 (unpaired, two-tailed Student’s t-test). (TIF) [file ppat.1011742.s002.tif]

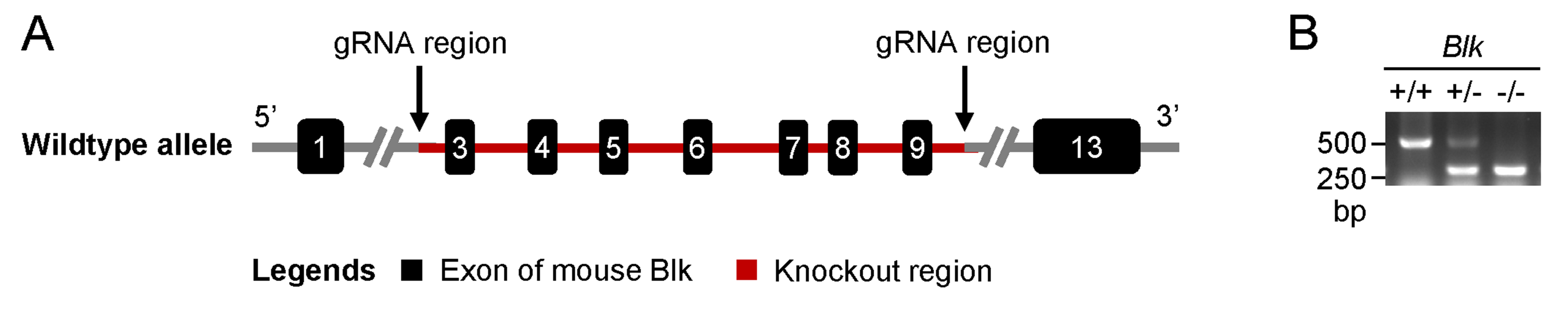

Supplement: S3 Fig — (A) Blk gene targeting strategy. The exon 3–9 and part of intron of Blk gene were deleted by CRISPR/Cas9 method. (B) Genotyping of Blk-/- mice. PCR analysis of genomic DNA to identify wild-type, heterogenous and Blk-/- mice. (TIF) [file ppat.1011742.s003.tif]

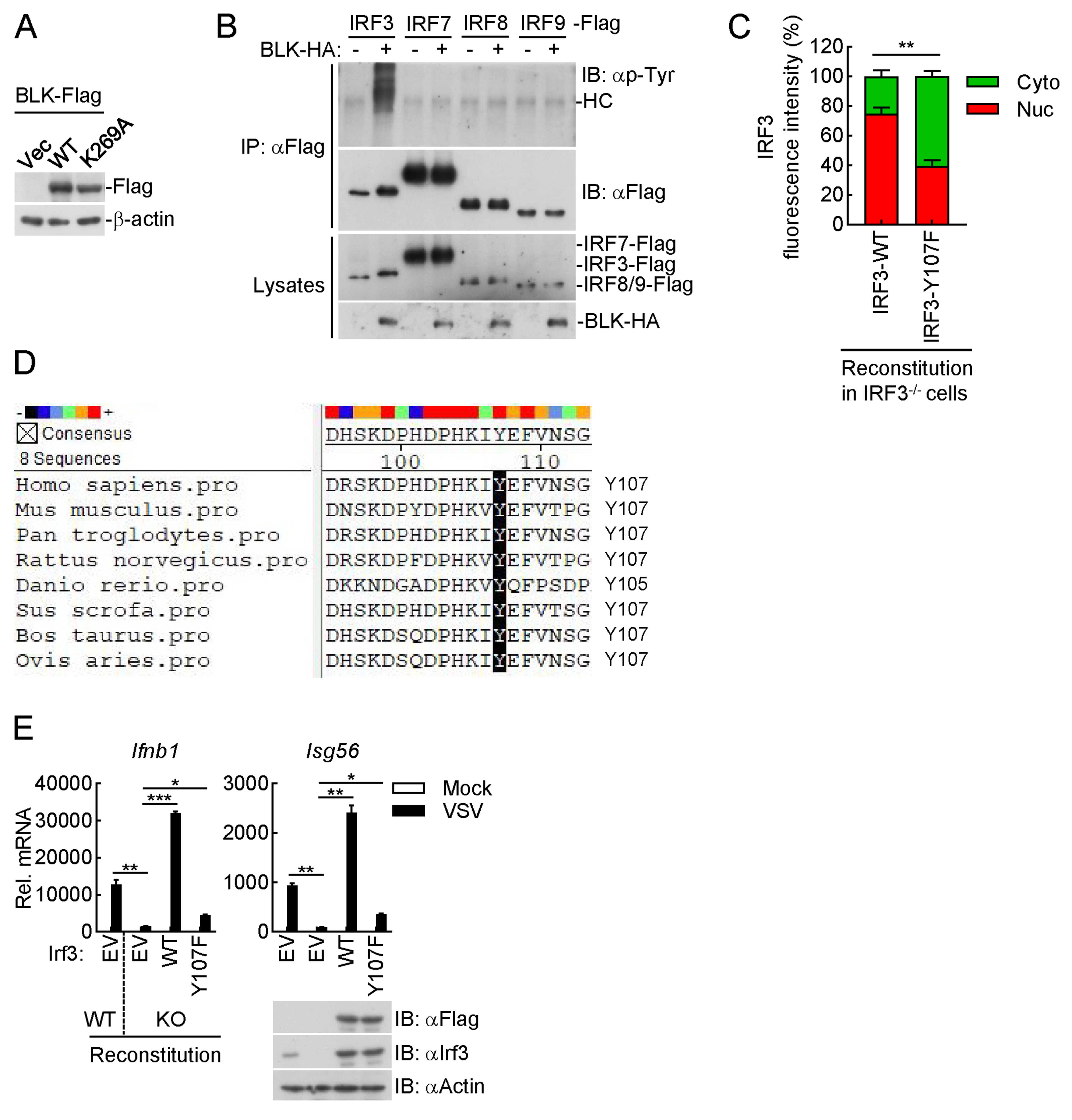

Supplement: S4 Fig — (A) The expression levels of BLK and BLK(K269A). U87MG cells (2 × 10^5^) were transfected with BLK or BLK(K269A) plasmids for 24 h. Cells were then harvested for immunoblot analysis. (B) BLK mediates tyrosine phosphorylation of IRF3. HEK293 cells (2 × 10^6^) were transfected with the indicated plasmids for 24 h. Coimmunoprecipitation and immunoblot analysis were performed with the indicated antibodies. (C) Quantitative analysis of IRF3 fluorescence intensity in the cytoplasm and nucleus in Fig 6G. Statistical analyses were based on images (covering dozens of cells) using ImageJ software. (D) Sequence alignment of IRF3 from the indicated species. The sequences correspond to aa95-113 of human IRF3. The conserved tyrosine residues are highlighted in black. (E) Effects of Irf3 or Irf3(Y107F) recovery on VSV-induced transcription of downstream antiviral genes. A20 cells were transduced with gRNA plasmids targeting murine Irf3 by the CRISPR/Cas9 method to establish the stable cell lines with puromycin (1 μg/mL) selection. Wild-type and Irf3-deficient A20 cells were transduced with empty vector (EV), Irf3, or Irf3(Y107F) plasmids by lentivirus-mediated gene transfer to establish the stable cell lines with blasticidin S (10 μg/mL) selection. The indicated cell lines (2 × 10^5^) were then uninfected or infected with VSV (MOI, 1) for 8 h before qPCR analysis. The lower blots show the expression levels of Irf3 and Irf3(Y107F) in the indicated cell lines as detected by anti-Flag or anti-Irf3 antibodies, respectively. Graphs show mean ± SD (n = 12 cells from three individual images in C, n = 2 technical replicates in E) from one representative experiment. Data are representative of at least three independent experiments with similar results. *P < 0.05, **P < 0.01, ***P < 0.001 (unpaired, two-tailed Student’s t-test). (TIF) [file ppat.1011742.s004.tif]

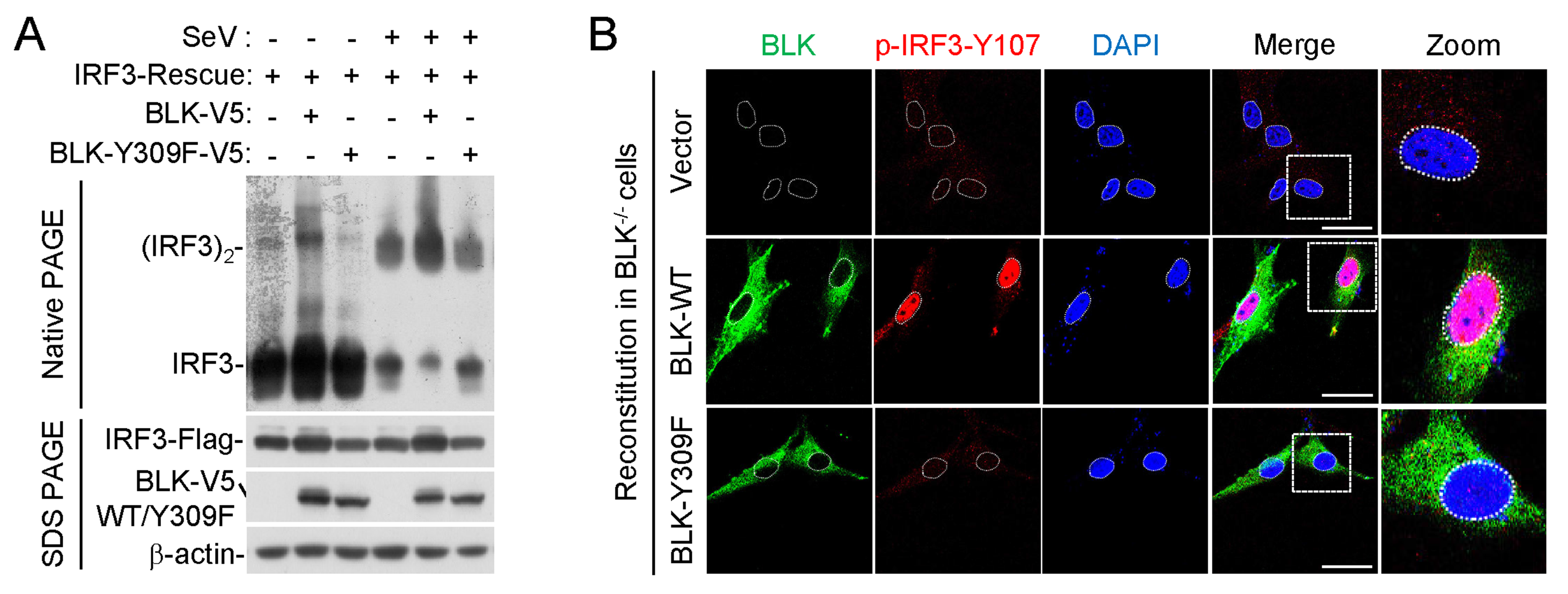

Supplement: S5 Fig — (A) Effects of BLK or BLK(Y309F) on SeV-triggered IRF3 dimerization. IRF3-reconstituted U87MG cells (5 × 10^5^) were transfected with the same amount of BLK or BLK(Y309F) plasmids for 24 h. Cells were then uninfected or infected with SeV (MOI, 1) for 6 h before native PAGE and SDS PAGE analyses. (B) Effects of BLK or BLK(Y309F) recovery on SeV-triggered IRF3 Y107 phosphorylation. BLK- or BLK(Y309F)-reconstituted U87MG cells (1 × 10^5^) were infected with SeV (MOI, 1) for 6 h, then fixed with 4% paraformaldehyde and stained with anti-Flag and anti-phospho-IRF3 (Y107) antibodies before confocal microscopy. Scale bars, 50 μm. Data are representative of at least three independent experiments with similar results. (TIF) [file ppat.1011742.s005.tif]
